# Modulation of Cd carriers by innovative nanocomposite (Ca+Mg) and Cd-resistance microbes (*Bacillus pumilus*): a mechanistic approach to enhance growth and yield of rice (*Oryza sativa* L.)

**DOI:** 10.3389/fpls.2024.1387187

**Published:** 2024-09-03

**Authors:** Muhammad Azhar Ali, Muhammad Nafees, Muhammad Waseem, Sarah Owdah Alomrani, Khalid A. Al-Ghanim, Mohammed Ali Alshehri, Hao Zheng, Shafaqat Ali, Fengmin Li

**Affiliations:** ^1^ Institute of Coastal Environmental Pollution Control, Ministry of Education Key Laboratory of Marine Environment and Ecology, and College of Environmental Science and Engineering, Ocean University of China, Qingdao, China; ^2^ State Key Laboratory of Pollution Control and Resource Reuse, School of the Environment, Nanjing University, Nanjing, China; ^3^ Department of Environmental Sciences and Engineering, Government College University, Faisalabad, Pakistan; ^4^ Department of Biology, College of Science and Arts, Najran University, Najran, Saudi Arabia; ^5^ Department of Zoology, College of Science, King Saud University, Riyadh, Saudi Arabia; ^6^ Department of Biology, Faculty of Science, University of Tabuk, Tabuk, Saudi Arabia; ^7^ Institute of Coastal Environmental Pollution Control, Key Laboratory of Marine Environment and Ecology, Ministry of Education, Frontiers Science Center for Deep Ocean Multispheres and Earth System, Ocean University of China, Qingdao, China; ^8^ Department of Biological Sciences and Technology, China Medical University, Taichung, Taiwan

**Keywords:** calcium, magnesium, nanocomposite, rice, *Bacillus pumilus*

## Abstract

Cadmium (Cd) is a well-known pollutant in agricultural soil, affecting human health through the food chain. To combat this issue, Ca + Mg (25 mg L^−1^) nanocomposite and *Bacillus pumilus*, either alone or combined, were applied to rice plants under Cd (5 mg kg^−1^, 10 mg kg^−1^) contamination. In our study, growth and yield traits demonstrated the beneficial influence of Ca + Mg and *B. pumilus* application in improving rice defense mechanism by reducing Cd stress. Combined Ca + Mg and *B. pumilus* application increased SPAD (15), total chlorophyll (18), chlorophyll a (11), chlorophyll b (22), and carotenoids (21%) with Cd (10 mg kg^−1^), compared to the application alone. Combined Ca + Mg and *B. pumilus* application significantly regulated MDA (15), H_2_O_2_ (13), EL (10), and O_2_^•–^ (24%) in shoots under Cd (10 mg kg^−1^), compared to the application alone. Cd (10 mg kg^−1^) increased the POD (22), SOD (21), APX (12), and CAT (13%) in shoots with combined Ca + Mg and *B. pumilus* application, compared to the application alone. Combined Ca + Mg and *B. pumilus* application significantly reduced Cd accumulation in roots (22), shoots (13), and grains (20%) under Cd (10 mg kg^−1^), compared to the application alone. Consequently, the combined application of Ca + Mg and *B. pumilus* is a sustainable solution to enhance crop production under Cd stress.

## Graphical Abstract

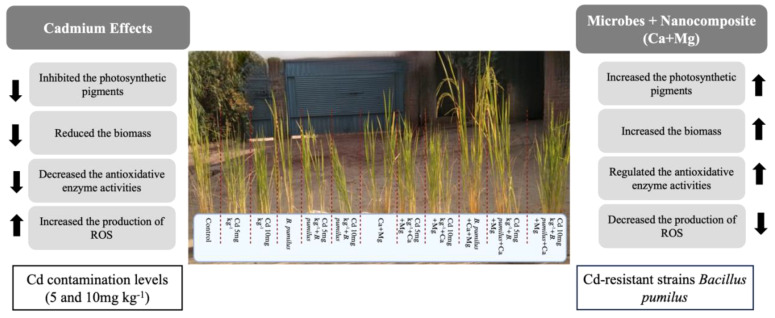

## Highlights

- Cd toxicity inhibited rice growth, yield, and antioxidative activity more than control.
- Cd toxicity decreased the nutrient uptake by increasing Cd accumulation.
- Ca + Mg + *B. pumilus* application enhanced rice growth and regulated the oxidative stress, compared to the application alone.
- Ca + Mg + *B. pumilus* application reduced Cd uptake and increased macro and micronutrients in shoots and grains, compared to the application alone.

## 1 Introduction

Heavy metal contamination, such as cadmium (Cd), is a widespread environmental hazard that has gained significant attention in agricultural soil (Alengebawy et al., 2021; Ali et al., 2022a; Zulfiqar et al., 2023a). Cd is rated 7th on the list of hazardous compounds due to its carcinogenic properties, according to the US Agency for Toxic Substances and Disease Registry (ASTDR, 2021; Zulfiqar et al., 2022a). Due to inadequate crop production strategies, Cd has caused food safety issues in recent years. Plants rapidly uptake, accumulate, and translocate Cd into different parts, particularly edible ones, triggering food safety issues (Liu et al., 2010; Zeb et al., 2022; Zulfiqar et al., 2022b). Rice is a staple crop with an annual production of 754.6 million tons, feeding half of the world’s population and representing a vital component of the global agricultural economy (FAO, 2017). Cd toxicity inhibits plant height, root length, leaf area, and the number of leaves per plant in rice (Song et al., 2015). Cd significantly reduces the growth and agronomical attributes of rice (Wang et al., 2014; Li et al., 2022). Numerous studies reported that Cd stress enhances oxidative stress through reactive oxygen species (ROS) production in rice seedlings (Srivastava et al., 2014; Ayyaz et al., 2022; Nazir et al., 2022). Therefore, it is essential to develop an effective remediation strategy to deal with Cd-contaminated soil. Various physical, chemical, and biological remediation techniques have been adopted for the removal of heavy metal contamination. These techniques are often unrealistic due to poor efficiency (Nafees et al., 2018; Hou et al., 2020; Yang et al., 2020). Hence, there is a need to develop cost-effective, eco-friendly, and innovative approaches to limit the impact of heavy metal pollution on global food safety (Chen and Li, 2018; Li et al., 2022).

Nanotechnology has garnered significant attention in the modern era, as this field has radically transformed modern science and is expanding exponentially (Bhardwaj et al., 2022). Recently, nanoparticles (NPs) have significantly enhanced plant growth and development by reducing heavy metal uptake in plants (Nafees et al., 2020; Babu et al., 2022; Ulhassan et al., 2022; Khalid et al., 2023; Nafees et al., 2024a). NPs mitigate stress by regulating phytohormones (Maqsood et al., 2023). According to a study, copper (Cu) NPs enhanced the growth and agronomical attributes of wheat by mitigating Cd toxicity (Noman et al., 2020). The foliar application of nano zinc (Zn) and iron (Fe) has shown a beneficial effects in *Rosmarinus officinalis* (Hassanpouraghdam et al., 2020). Similarly, the foliar application of ceric oxide (CeO_2_) and copper oxide (CuO) NPs enhanced the fresh weight, yield, and nutritional quality of cucumber (Hong et al., 2016).

Calcium (Ca) and magnesium (Mg) are ubiquitous metal elements in the Earth’s crust, and their ionic forms (Ca^2+^ and Mg^2+^) in soil are vital plant nutrients. Mg plays a fundamental role in regulating physical and biochemical processes, and its deficiency is a limiting factor for crop production (Ishfaq et al., 2021, 2022). Ca^2+^ and Mg^2+^ are absorbed by roots and transported to shoots via xylem. CaO NPs significantly enhanced plant biomass, and enzymatic and non-enzymatic antioxidative activity due to a substantial decrease in ROS species under Cd toxicity (Nazir et al., 2022). Li et al. (2022) reported that Ca^2+^ and Mg^2+^ treatment boosted rice growth and yield by reducing uptake and accumulation of Cd in grains, roots, stems, and leaves. Limited research has focused on investigating the influence of Ca and Mg NPs on plant–soil systems, particularly regarding soil microbial interactions with plants.

Like NPs, microbes play a vital role in plant growth by reducing the uptake and accumulation of heavy metals (Hassan et al., 2017; Ali et al., 2022b; Zulfiqar et al., 2023b). *Bacillus pumilus* is a growth-promoting bacterium in plants. *B. pumilus* reduces the uptake of cadmium and promotes plant height and photosynthetic pigments in rapeseed (*Brassica napus* L.) (Masood et al., 2020). Similarly, *Bacillus* spp. enhances the activity of ROS-scavenging enzymes such as POD, SOD, CAT, and APX, and boosts maize tolerance to Zn and Cu stress (Shahzad et al., 2021). Currently, no studies have been conducted on the combined effect of Ca + Mg nanocomposite and microorganisms on rice growth. Furthermore, the risk assessment of their toxicity to rice and soil is still in its early stages. Meanwhile, the combination of nanoparticles and microbial strain inoculation has recently attracted significant attention in agriculture due to their greater efficacy in alleviating heavy metal stress (Babu et al., 2022; Ahmed et al., 2023).

Therefore, a novel Ca + Mg nanocomposite was synthesized and the specific objectives of the current study were: a) to evaluate the individual and combined effect of Ca + Mg nanocomposite and *B. pumilus* on rice yield and growth; b) investigate the alleviating combined role of the Ca + Mg nanocomposite and microbes on oxidant and antioxidant enzymatic activity; c) to analyze the individual and combined effect of the Ca + Mg nanocomposite and *B. pumilus* on Cd uptake and accumulation in rice. Thus, the current study could offer an economically feasible alternative fertilizer that promotes sustainable agricultural crop production with higher nutritional value.

## 2 Material and methods

### 2.1 Soil collection and scrutiny

The soil was collected from the fields of the University of Agriculture, Faisalabad. Soil was air-dried and sieved through a 2 mm sieve. The soil used in this study was sandy clay loam according to Bouyoucos (1962). The electrical conductivity (EC) was 1.85 dS m^−1^, and the pH (7.68) of the soil extract was measured using appropriate methods. Available Cd (0.07 mg kg^−1^) was measured following the standard procedure of Amacher (1996). Soil organic carbon was assessed by following the Walkley–Black protocol, while calcium carbonate, total nitrogen, available phosphorous, extractable potassium, and cation exchange capacity were 3.16 g kg^−1^, 3.2%, 0.089%, 6.1 mg kg^−1^, 92 mg kg^−1^, 10.4 cmol_(+)_ kg^−1^, respectively, using appropriate methods. Similarly, Zn, Mn, and Fe were 5.13 mg kg^−1^, 4.97 mg kg^−1^, and 53.74 mg kg^−1^, respectively, determined using the calcimeter method (Moodie et al., 1959; Jackson, 1962).

### 2.2 Ca + Mg nanocomposite synthesis

**Figure 1:**
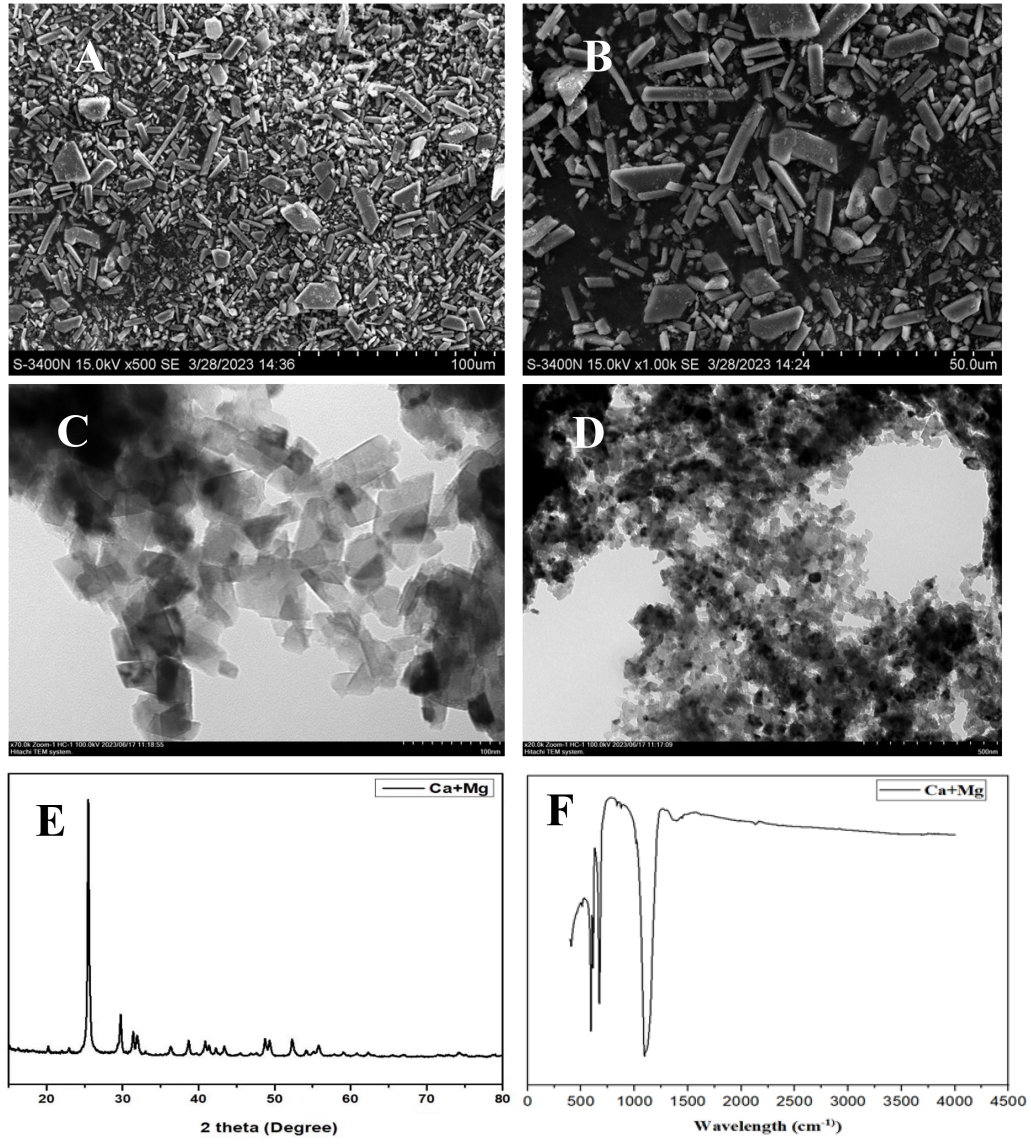
SEM of calcium and magnesium (Ca+Mg) **(A, B)**, TEM **(C, D)**, XRD **(E)** and FTIR **(F)**.

The calcium and magnesium nanocomposite was synthesized following the method of Das et al. (2018) using calcium nitrate tetrahydrate (Ca(NO_3_)_2_ · 4H_2_O) and magnesium nitrate tetrahydrate (Mg(NO_3_)_2_ · 4H_2_O). Separate solutions of calcium nitrate tetrahydrate (0.1 M) and magnesium nitrate tetrahydrate (0.1 M) were prepared. Both solutions were mixed, and sodium hydroxide (NaOH) pellets were added to maintain a pH 11.5. The mixture was stirred on a hotplate at 90°C, 120 g for 6 h. The formation of the Ca and Mg nanocomposite was confirmed by visually observing the color change of the solution. After 6 h, the solution was cooled to room temperature and centrifuged at 7,000*g*, 25°C for 5 min. The obtained residue was then washed several times with double-distilled water, dried in an oven at 80°C for 24 h, and ground using a mortar and pestle. The nanocomposite was calcined at 500°C for 3 h using a furnace tube to homogenize and remove impurities. X-ray diffraction (XRD) patterns and Fourier transform infrared (FT-IR) spectra were analyzed using PANalytical B·V. (Netherlands) and PerkinElmer, respectively. The surface morphology of the nanocomposite was examined by scanning electron microscopy coupled with energy-dispersive X-ray spectroscopy (SEM-EDX) and transmission electron microscopy (TEM), as shown in **Figure 1**.

### 2.3 Seeds inoculation with *B. pumilus*

The isolated strain *B. pumilus* (KF859972) was collected from the Department of Microbiology, GC University, Faisalabad, and prepared following Zeng et al. (2020). Nutrient broth (250 mL) simmered on a rotary shaker (150 g, 37°C, 24 h), then collected after centrifugation (10,000*g*, 10 min). The supernatant was discarded, and the residue was washed with sterilized distilled water. Surface-sterilized seeds (hydrogen peroxide 10% H_2_O_2_) were inoculated with bacteria using carboxymethyl cellulose (2%) on a rotary shaker at room temperature (90 g). A mixture of clay and peat moss (1:1 w/w) was used to coat the inoculated seeds. Both normal and inoculated seeds were planted in control and respective treatment conditions according to the treatment plan.

### 2.4 Experimental conditions

A pot experiment was carried out under natural environmental conditions (day–night temperature, 39/32°C, and humidity, 78 ± 4%) in 2022 at the Botanical Garden of Government College University Faisalabad, Pakistan. Treatments includes: Control; Cd 5 mg kg^−1^, Cd 10 mg kg^−1^, Microbes, Cd 5 mg kg^−1^ + Microbes, Cd 10 mg kg^−1^ + Microbes, (Ca + Mg) foliar, Cd 5 mg kg^−1^ + (Ca + Mg) foliar, Cd 10 mg kg^−1^ + (Ca + Mg) foliar, Microbes + (Ca + Mg) foliar, Cd 5 mg kg^−1^ + (Ca + Mg) foliar + Microbes, Cd 10 mg kg^−1^ + (Ca + Mg) foliar + Microbes. Pots were filled with sifted soil (5 kg pot^−1^) spiked with Cd (5 mg kg^−1^ and 10 mg kg^−1^) using cadmium chloride according to the treatment plan. A complete randomized design was used to conduct the experiment in triplicate. Rice seeds were soaked in water for 48 h, inoculated with *B. pumilus* before sowing, and grown in sieved and washed sand. After 20 days of germination, the rice plants were transplanted into pots, each containing four healthy plants. The recommended dose of NPK fertilizer was applied to prevent nutrient deficiency. The foliar Ca + Mg nanocomposite was applied after germination. A total of seven foliar applications were sprayed at one-and-a-half week intervals.

### 2.5 Measurement of photosynthetic pigments and gas exchange parameters

SPAD value was measured using an *in situ* SPAD meter. Photosynthetic pigments such as chlorophyll a and b, total chlorophyll, and carotenoids were determined spectrophotometrically in fresh rice leaves (Lichtenthaler, 1987). Acetone 85% (v/v ratio) was used to extract the samples to assess chlorophyll and carotenoid contents. Readings were taken using a spectrophotometer after extraction and centrifugation of samples. Gas exchange parameters (photosynthesis rate, transpiration rate, stomatal conductance, and water use efficiency) were measured during sunlight (12:00 a.m.) in leaves of rice plants using an infrared gas analyzer (IRGA).

### 2.6 Harvesting of plants

Plants were harvested and carefully separated into shoots, roots, leaves, and grains after 120 days of sowing. Growth parameters such as root and shoot lengths (cm), root fresh and dry weights (g), shoot fresh and dry weights (g), spike length, and number of grains were determined. Roots were washed with HCl (0.1%) and distilled water to remove metals. Root, shoot, and grain samples were oven-dried (72 h at 80°C) and crushed into small pieces for further analysis.

### 2.7 Measurement of oxidative stress and antioxidant enzymatic activity

Malondialdehyde (MDA) levels were assessed using the TBA (0.1%) method (Zhang and Kirkham, 1994; Abbas et al., 2017). Electrolyte leakage determination was achieved in two steps. Initial EC was noted after incubating samples for 2 h at 32°C. Second EC was measured for 20 min at 121°C following Dionisio-Sese and Tobita (1998). H_2_O_2_ activity was analyzed as per Jana and Choudhuri (1982). Samples were crushed in phosphate buffer (PB 5 Mm and pH 6.5) and centrifuged for 20 min. Sulfuric acid (20%) was added after centrifugation and then centrifuged for 15 min. Absorbance was measured at a wavelength of 410 nm using a spectrophotometer. Superoxide radical (O_2_^•–=^) contents were measured by obtaining fresh leaf extract in hydroxylamine hydrochloride, titrated with naphthylamine (7 mM) and sulfanilamide (17 mM) (Yang et al., 2011).

Phosphate buffer (PB 0.5, at pH 7.8) was used to homogenize the leaf and root samples for the determination of peroxidase (POD) and superoxide dismutase (SOD) contents (Zhang, 1992). Ascorbate peroxidase (APX) activity was estimated using the Nakano and Asada (1981) protocol, while CAT contents were assessed using the Aebi (1984) method.

### 2.8 Measurement of metabolites

Total soluble proteins (TSP) were measured by homogenizing 0.5 g of fresh leaves in potassium phosphate buffer (50 mM, pH 7.5) following Bradford (1976). Fresh leaves (0.5 g) were crushed in a potassium phosphate buffer solution (50 mM, pH 7.5). Pyridine and acid ninhydrin were used to titrate the supernatant to measure total free amino acids (TFAA) (Hamilton et al., 1943). Total soluble sugars (TSS) were analyzed by homogenizing 0.5 g of fresh leaves in an ethanol and ethanol mixture, and the extract was reacted with an anthrone reagent (Yemm and Willis, 1954). Phenolics were determined by triturating 0.5 g of fresh leaves in acetone, centrifuging at 10,000*g* for 10 min, and then reacting the supernatant with Folin and Ciocalteau’s phenol reagent for determination (Wolfe et al., 2003).

### 2.9 Assessment of metal contents and macro and micronutrients

The protocol of Lwalaba et al. (2020) was adopted with slight modification. Briefly, samples were digested in a diacid mixture of HNO_3_:HClO_4_ (4:1 v/v) at 140°C on a hotplate. Concentrations of elements such as Ca, Mg, Mn, Zn, Fe, K, and Cd were determined using ICP-MS (iCAP RQ, Thermo Scientific).

### 2.10 Statistical analysis

Data analysis was performed using SPSS version 16.0 (SPSS, Chicago, IL). One-way analysis of variance (ANOVA) was conducted, following the Tukey HSD test to assess significant differences among means.

## 3 Results

### 3.1 Effect of Ca + Mg nanocomposite and *B. pumilus* on growth parameters

Foliar application of Ca + Mg nanocomposite and microbial inoculation showed positive effects on rice growth, physiology, and antioxidant contents under Cd (5 mg kg^−1^, 10 mg kg^−1^) toxicity. The results showed that Cd (5 mg kg^−1^) reduced the length of roots and shoots by 20% and 30%, weight of fresh roots and shoots by 22% and 13%, weight of dry roots and shoots by 19% and 16%, number of tillers and grains by 38% and 19%, and spike length by 21% compared to the control treatment. Similarly, the roots and shoots length were reduced by 44% and 46%, weight of fresh roots and shoots by 42% and 32%, weight of dry roots and shoots by 36% and 37%, number of tillers and grains by 59% and 35%, and spike length by 41% significantly (*p* ≤*0.05*) inhibited with Cd (10 mg kg^−1^) compared with control treatment. Meanwhile, the *B. pumilus* significantly (*p* ≤*0.05*) increased the length of roots and shoots length by 23% and 22%, weight of fresh roots and shoots by 26% and 10%, wet of dry roots and shoots by 21% and 12%, number of tillers and grains by 20% and 19%, and spike length by 13% compared to the control. Moreover, *B. pumilus* inoculation enhanced the length of the roots and shoots by 3% and 12%, weight of fresh roots and shoots by 10% and 11%, weight of dry roots and shoots by 7% and 11%, number of tillers and grains by 46% and 16%, and spike length by 15% in Cd (5 mg kg^−1^) contamination compared without *B. pumilus* treatment. Under Cd (10 mg kg^−1^) contamination, *B. pumilus* improved the length of roots and shoots by 11% and 19%, weight of fresh roots and shoots by 15% and 19%, weight of dry roots and shoots by 9% and 25%, number of tillers and grains by 75% and 22%, and spike length by 24% compared without *B. pumilus* treatment.

Foliar application of Ca + Mg nanocomposite promoted the length of roots and shoots by 22 and 14%, weight of fresh roots and shoots by 23 and 10%, weight of dry roots and shoots by 18 and 15%, number of tillers and grains by 18 and 20%, and spike length by 26% compared to control treatment. Application of Ca + Mg nanocomposite boosted the length of roots and shoots by 17 and 15%, weight of fresh roots by 9%, weight of dry roots and shoots by 8 and 2%, number of grains by 4%, spike length by 4%, and decreased the weight of fresh shoots by 1% and number of tillers by 3% under Cd (5 mg kg^−1^) contamination compared to Cd (5 mg kg^−1^) + *B. pumilus* treatment. Similarly, Ca + Mg nanocomposite foliar application enhanced the length of roots and shoots by 21% and 18%, weight of fresh roots by 13%, dry roots and shoots weight 17% and 3%, number of grains by 3%, spike length by 4%, while decreasing the weight of fresh shoots by 2%, and no. of tillers by 7% under Cd (10 mg kg^−1^) contamination compared with Cd (10 mg kg^−1^) + *B. pumilus* treatment.

The highest increase was observed with the combined application of Ca + Mg foliar spray and *B. pumilus*. Significantly (*p* ≤*0.05*), the combined application of Ca + Mg and *B. pumilus* inoculation boosted length of roots and shoots by 14% and 8%, weight of fresh roots and shoots by 11% and 9%, weight of dry roots and shoots by 8% and 10%, number of tillers and grains by 21% and 16%, and spike length by 21% compared with *B. pumilus* treatment. While, the combined application of Ca + Mg and *B. pumilus* inoculation augmented the length of roots and shoots by 31% and 28%, weight of fresh roots and shoots by 17% and 9%, weight of dry roots and shoots by 15% and 14%, number of tillers and grains by 17% and 19%, and spike length by 23% with Cd (5 mg kg^−1^) contamination as compared to Cd (5 mg kg^−1^) + *B. pumilus* treatment. Similarly, the combined application of Ca + Mg and *B. pumilus* significantly (*p* ≤*0.05*) boosted the length of roots and shoots by 43% and 32%, weight of fresh roots and shoots by 27% and 16%, weight of dry roots and shoots by 24 and 14%, number of tillers and grains by 25% and 22%, and spike length by 29% under Cd (10 mg kg^−1^) as compared with Cd (10 mg kg^−1^) + *B. pumilus* treatment.

### 3.2 Effect of Ca + Mg nanocomposite and *B. pumilus* on photosynthetic pigments

The statistical analysis showed that the SPAD values, chlorophyll a, b, total chlorophyll, and carotenoid with Cd (5 mg kg^−1^, 10 mg kg^−1^) toxicity decreased at a significant (*p* ≤*0.05*). Results showed that the Cd (5 mg kg^−1^) abridged the SPAD values, chlorophyll a, b, total chlorophyll, and carotenoid contents 12%, 21%, 32%, 25%, and 33%, compared with the control treatment. Similarly, Cd (10 mg kg^−1^) at a significant (*p* ≤*0.05*) minimized the SPAD, chlorophyll a, b, total chlorophyll, and carotenoid contents 34%, 36%, 49%, 42%, and 55%, as compared to the control treatment. Besides, compared with the control treatment, *B. pumilus* application boosted SPAD values, chlorophyll a, b, total chlorophyll, and carotenoids 23%, 17%, 13%, 15%, and 17%. Similarly, *B. pumilus* enhanced the SPAD, chlorophyll a, b, total chlorophyll, and carotenoid contents 6%, 12%, 25%, 26%, and 25% with Cd (5 mg kg^−1^) contamination as compared with respective treatment. Under Cd (10 mg kg^−1^) stress, *B. pumilus* inoculation at a significant (*p* ≤*0.05*) enhanced the SPAD, chlorophyll a, b, total chlorophyll, and carotenoid contents 17%, 20%, 33%, 26%, and 50% compared with respective treatment.

Ca + Mg nanocomposite increased the SPAD, chlorophyll a, b, total chlorophyll, and carotenoid contents by 10%, 9%, 8%, 10%, and 12% as compared with control. Under Cd (5 mg kg^−1^) contamination, Ca + Mg nanocomposite enhanced the SPAD, chlorophyll a, b, total chlorophyll, and carotenoid contents 5%, 9%, 2%, 5%, and 10% as compared with Cd (5 mg kg^−1^) + *B. pumilus* treatment. Similarly, Ca + Mg nanocomposite application increased the SPAD values, chlorophyll a, b, total chlorophyll, and carotenoid contents by 9%, 6%, 8%, 6%, and 12% with Cd (10 mg kg^−1^) contamination as compared with Cd (10 mg kg^−1^) + *B. pumilus* treatment. Besides this, combined application of microbial inoculation and Ca + Mg nanocomposite significantly enhanced the SPAD, chlorophyll a, b, total chlorophyll, and carotenoid contents. The results showed that the combined Ca + Mg and *B. pumilus* application considerably increased the SPAD, chlorophyll a, b, total chlorophyll, and carotenoid contents 9%, 10%, 15%, 18%, and 11% as compared with *B. pumilus* treatment. However, the combined Ca + Mg and *B. pumilus* application increased the SPAD, chlorophyll a, b, total chlorophyll, and carotenoid contents by 13%, 16%, 15%, 17%, and 20% with Cd (5 mg kg^−1^) contamination as compared with Cd (5 mg kg^−1^) + *B. pumilus* treatment. Similarly, Cd (10 mg kg^−1^) contamination with combined Ca + Mg and *B. pumilus* application at a significant (*p* ≤*0.05*) increased the SPAD, chlorophyll a, b, total chlorophyll, and carotenoid contents 15%, 11%, 22%, 18%, and 21% as compared with Cd (10 mg kg^−1^) + *B. pumilus* treatment.

Likewise, the SPAD, chlorophyll, and carotenoid contents, the photosynthesis rate (Pn), transpiration rate (Tr), water use efficiency (WUE), and stomatal conductance were also enhanced with foliar application of Ca + Mg nanocomposite and microbial inoculation under Cd (5 mg kg^−1^, 10 mg kg^−1^) stress. Results showed that the Pn, Tr, WUE, and stomatal conductance were inhibited 20%, 23%, 22%, and 24% with Cd (5 mg kg^−1^) contamination, as compared with control. Similarly, under Cd (10 mg kg^−1^), the Pn, Tr, WUE, and stomatal conductance were reduced 44%, 39%, 42%, and 40%, respectively, compared with the control. While *B. pumilus* inoculation enhanced the Pn, Tr, WUE, and stomatal conductance 12%, 24%, 19%, and 26%, compared with the control. Moreover, *B. pumilus* treatment enhanced the Pn, Tr, WUE, and stomatal conductance 16%, 17%, 21%, and 20% with Cd (5 mg kg^−1^) contamination, compared to without *B. pumilus*. Under Cd (10 mg kg^−1^) contamination, *B. pumilus* inoculation at a significant (*p* ≤*0.05*) amplified the Pn, Tr, WUE, and stomatal conductance by 26%, 21%, 33%, and 27%, compared to without *B. pumilus* inoculation. Ca + Mg nanocomposite promoted the Pn, Tr, WUE, and stomatal conductance by 11%, 13%, 10%, and 18% as compared with control. Ca + Mg nanocomposite increased Pn, Tr, WUE, and stomatal conductance by 2%, 5%, 3%, and 9% with Cd (5 mg kg^−1^) contamination compared with Cd (5 mg kg^−1^) + *B. pumilus* treatment. Similarly, foliar application of Ca + Mg nanocomposite enhanced the Pn, Tr, WUE, and stomatal conductance 6%, 3%, 5%, and 1% with Cd (10 mg kg^−1^) contamination, compared with Cd (10 mg kg^−1^) + *B. pumilus* treatment.

The highest increase was observed with the combined application of Ca + Mg and *B. pumilus* inoculation. Further, the combined application of Ca + Mg and *B. pumilus* inoculation enhanced the Pn, Tr, WUE, and stomatal conductance by 17%, 9%, 11%, and 12% at a significant (*p* ≤*0.05*) compared to *B. pumilus* treatment. Meanwhile, combined Ca + Mg and *B. pumilus* application increased the Pn, Tr, WUE, and stomatal conductance by 16%, 11%, 13%, and 19% with Cd (5 mg kg^−1^) contamination compared with Cd (5 mg kg^−1^) + *B. pumilus* treatment. Correspondingly, the combined Ca + Mg and *B. pumilus* application significantly (*p* ≤*0.05*) promoted the Pn, Tr, WUE, and stomatal conductance by 28%, 10%, 19%, and 16% with Cd (10 mg kg^−1^) contamination compared with Cd (10 mg kg^−1^) + *B. pumilus* treatment.

### 3.3 Effect of Ca + Mg nanocomposite and *B. pumilus* on oxidative stress markers and antioxidant enzymatic activities

**Figure 2:**
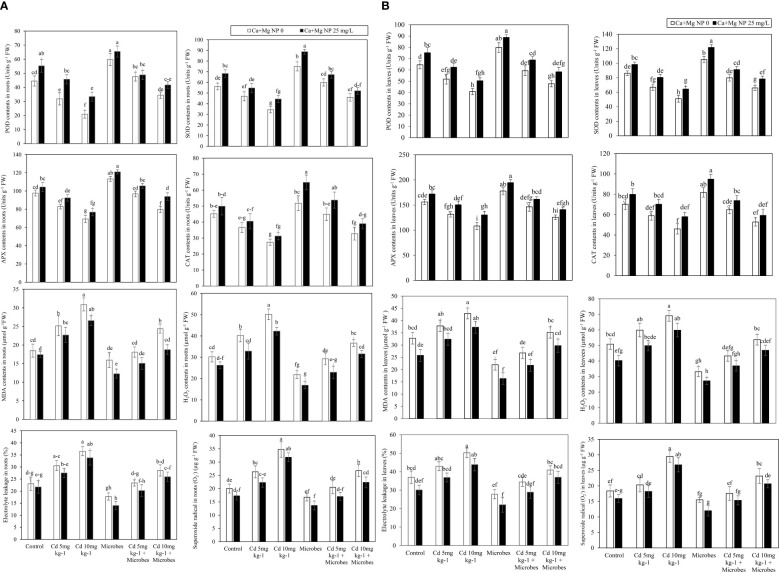
**(A)** Alone and combined effect of Ca+Mg nanocomposite (25 mg L^-1^) and inoculation of *Bacillus pumilus* on enzymatic antioxidants POD, SOD, APX and CAT and oxidants EL, MDA, H_2_O_2_ in roots and small letter showed the difference in significance at *p* ≤ 0.05 level with mean of three replications. **(B)** Alone and combined effect of Ca+Mg nanocomposite (25 mg L^-1^) and inoculation of *Bacillus pumilus* on enzymatic antioxidants POD, SOD, APX and CAT and oxidants EL, MDA, H_2_O_2_ in shoots and small letter showed the difference in significance at *p* ≤ 0.05 level with mean of three replications.

Foliar application of Ca + Mg nanocomposite and microbial inoculation showed a significant (*p* ≤*0.05*) effect on oxidants and antioxidants enzymatic activity in roots and leaves such as MDA, H_2_O_2_, EL, O_2_^•–^, POD, SOD, APX, and CAT with Cd (5 mg kg^−1^, 10 mg kg^−1^) contamination (**Figures 2A, B**). Results showed that Cd (5 mg kg^−1^) boosted MDA, H_2_O_2_, EL, and O_2_^•–^ in roots by 36%, 30%, 33%, and 31% and leaves by 15%, 18%, 16%, and 32% compared with control. Meanwhile, Cd (5 mg kg^−1^) declined the POD, SOD, APX, and CAT in roots by 28%, 16%, 15%, and 19% and leaves by 20%, 22%, 16%, and 17% compared with control. Similarly, Cd (10 mg kg^−1^) further enhanced the MDA, H_2_O_2_, EL, and O_2_^•–^ in roots by 67%, 63%, 58%, and 85% and leaves by 31%, 36%, 35%, and 91%, compared with control. The POD, SOD, APX, and CAT activity in roots by 53%, 39%, 29%, and 39% and leaves by 37%, 41%, 31%, and 34% decreased significantly (*p* ≤*0.05*) with Cd (10 mg kg^−1^) compared with control. Meanwhile, *B. pumilus* inoculation abridged the MDA, H_2_O_2_, EL, and O_2_^•–^ contents in roots by 14%, 29%, 23%, and 16% and leaves by 33%, 35%, 25%, and 16% compared with control.

*B. pumilus* inoculation at a significant (*p* ≤*0.05*) boosted POD, SOD, APX, and CAT activity in roots by 34%, 35%, 16%, and 15% and leaves by 24%, 22%, 14%, and 17% compared with control. Moreover, *B. pumilus* inoculation diminished the MDA, H_2_O_2_, EL, and O_2_^•–^ contents in roots by 28%, 27%, 22%, and 22% and leaves by 29%, 28%, 20%, and 25% with Cd (5 mg kg^−1^) contamination compared without *B. pumilus* inoculation. *B. pumilus* inoculation enhanced the POD, SOD, APX, and CAT in roots by 50%, 28%, 17%, and 22% and leaves by 14%, 19%, 11%, and 10% with Cd (5 mg kg^−1^) contamination, compared to without *B. pumilus* treatment. Under Cd (10 mg kg^−1^) contamination, the *B. pumilus* inoculation significantly (*p* ≤*0.05*) inhibited MDA, H_2_O_2_, EL, and O_2_^•–^ production in roots by 21%, 27%, 22%, and 21% and leaves by 18%, 22%, 19%, and 24% compared without *B. pumilus* treatment. Similarly, *B. pumilus* inoculation significantly (*p* ≤*0.05*) increased POD, SOD, APX, and CAT activity in roots by 65%, 34%, 15%, and 19% and leaves by 17%, 29%, 16%, and 15% under Cd (10 mg kg^−1^) contamination compared without *B. pumilus* treatment.

Ca + Mg nanocomposite further reduced the production of MDA, H_2_O_2_, EL, and O_2_^•–^ in roots by 5%, 15%, 6%, and 14% and leaves by 21%, 20%, 18%, and 13% as compared with control. Ca + Mg nanocomposite boosted the POD, SOD, APX, and CAT in roots by 24%, 21%, 7%, and 11% and leaves by 17%, 14%, 10%, and 14% as compared with control. Ca + Mg nanocomposite diminished the MDA, H_2_O_2_, EL, and O_2_^•–^ production in roots by 25%, 12%, 17%, and 8% and leaves by 21%, 15%, 7%, and 15% with Cd (5 mg kg^−1^) contamination compared with Cd (5 mg kg^−1^) + *B. pumilus* treatment. Similarly, foliar application of Ca + Mg nanocomposite increased the POD, SOD, APX, and CAT in roots by 4%, 9%, 5%, and 10% and leaves by 5%, 1%, 3%, and 9% with Cd (5 mg kg^−1^) contamination compared with Cd (5 mg kg^−1^) + *B. pumilus* treatment. Similarly, Ca + Mg nanocomposite lessened the production of MDA, H_2_O_2_, EL, and O_2_^•–^ in roots by 9%, 15%, 18%, and 9% and leaves by 7%, 11%, 10%, and 13% with Cd (10 mg kg^−1^) contamination compared with Cd (10 mg kg^−1^) + *B. pumilus* treatment. Additionally, Ca + Mg nanocomposite foliar application promoted the POD, SOD, APX, and CAT in roots by 3%, 4%, 4%, and 5% and leaves by 6%, 2%, 4%, and 10% with Cd (10 mg kg^−1^) contamination compared with Cd (10 mg kg^−1^) + *B. pumilus* treatment.

The highest increase was observed with combined Ca + Mg and *B. pumilus* application. Further, the combined application of Ca + Mg foliar spray and *B. pumilus* inoculation significantly (*p* ≤*0.05*) inhibited MDA, H_2_O_2_, EL, and O_2_^•–^ production in roots by 23%, 22%, 21%, and 19% and leaves by 26%, 18%, 21%, and 23% compared with *B. pumilus* treatment. The combined Ca + Mg foliar spray and *B. pumilus* inoculation augmented the POD, SOD, APX, and CAT activity in roots by 9%, 18%, 7%, and 25% and leaves by 11%, 16%, 9%, and 16% as compared with *B. pumilus* treatment. Combined Ca + Mg foliar spray and *B. pumilus* inoculation inhibited the MDA, H_2_O_2_, EL, and O_2_^•–^ in roots by 17%, 22%, 14%, 17%, and leaves by 19%, 15%, 17%, and 16% with Cd (5 mg kg^−1^) contamination as compared with Cd (5 mg kg^−1^) + *B. pumilus* treatment. The combined Ca + Mg foliar spray and *B. pumilus* inoculation increased the POD, SOD, APX, and CAT in roots by 2%, 12%, 9%, and 20% and leaves by 16%, 13%, 10%, and 14% with Cd (5 mg kg^−1^) contamination compared with Cd (5 mg kg^−1^) + *B. pumilus* treatment. Similarly, the combined Ca + Mg foliar spray and *B. pumilus* inoculation further decreased the MDA, H_2_O_2_, EL, and O_2_^•–^ in roots by 23%, 14%, 9%, and 23% and leaves by 15%, 13%, 10%, and 24% with Cd (10 mg kg^−1^) as compared with Cd (10 mg kg^−1^) + *B. pumilus* treatment. The combined Ca + Mg foliar spray and *B. pumilus* inoculation enhanced the POD, SOD, APX, and CAT in roots by 21%, 13%, 18%, and 19% and leaves by 22%, 21%, 12%, and 13% with Cd (10 mg kg^−1^) compared with Cd (10 mg kg^−1^) + *B. pumilus* treatment.

### 3.4 Effect of nanocomposite Ca + Mg and *B. pumilus* on metabolites

**Figure 3:**
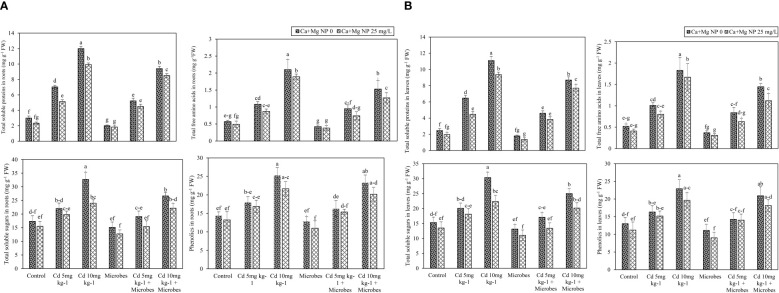
**(A)** Alone and combined effect of Ca+Mg nanocomposite (25 mg L^-1^) and inoculation of *Bacillus pumilus* on total soluble proteins, total free amino acids, total soluble sugars and phenolics in roots and small letter showed the difference in significance at *p* ≤ 0.05 level with mean of three replications. **(B)** Alone and combined effect of Ca+Mg nanocomposite (25 mg L^-1^) and inoculation of *Bacillus pumilus* on total soluble proteins, total free amino acids, total soluble sugars and phenolics in leaves and small letter showed the difference in significance at *p* ≤ 0.05 level with mean of three replications.

Foliar application of Ca + Mg nanocomposite and microbial inoculation showed a slight (*p* ≤*0.05*) effect on metabolites in roots and leaves such as TSP, TFAA, TSS, and phenolics under Cd (5 mg kg^−1^, 10 mg kg^−1^) toxicity (**Figures 3A, B**). Results demonstrated that Cd (5 mg kg^−1^, 10 mg kg^−1^) considerably (*p* ≤*0.05*) boosted TSP, TFAA, TSS, and phenolics in roots and leaves compared with the control treatment. Application of Ca + Mg nanocomposite and microbial inoculation slightly decreased the metabolites in roots and leaves with Cd (5 mg kg^−1^, 10 mg kg^−1^) contamination. Meanwhile, *B. pumilus* inoculation slightly (*p* ≤*0.05*) reduced the TSP, TFAA, TSS, and phenolics contents in roots by 33%, 27%, 12%, and 11% and leaves by 27%, 29%, 14%, and 15% as compared with control. Moreover, *B. pumilus* inoculation decreased the TSP, TFAA, TSS, and phenolics contents in roots by 11%, 19%, 13%, and 10% and leaves by 23%, 21%, 18%, and 13% with Cd (5 mg kg^−1^) contamination compared without *B. pumilus* treatment. Under Cd (10 mg kg^−1^) stress, *B. pumilus* inoculation slightly (*p* ≤*0.05*) inhibited the production of TSP, TFAA, TSS, and phenolics in roots by 9%, 27%, 13%, and 6% and leaves by 13%, 31%, 8%, and 5% compared without *B. pumilus* treatment. Ca + Mg nanocomposite reduced the TSP, TFAA, TSS, and phenolics in roots by 22%, 15%, 11%, and 8% and leaves by 20%, 21%, 12%, and 14%, compared to the control treatment. Ca + Mg nanocomposite increased the TSP, TFAA, TSS, and phenolics in roots by 3%, 9%, 4%, and 3% and leaves by 3%, 5%, 4%, and 7% with Cd (5 mg kg^−1^) contamination compared with Cd (5 mg kg^−1^) + *B. pumilus* treatment. Similarly, Ca + Mg nanocomposite boosted the TSP, TFAA, TSS, and phenolics in roots by 2%, 23%, 11%, and 7% and leaves by 4%, 31%, 3%, and 3% with Cd (10 mg kg^−1^) contamination compared with Cd (10 mg kg^−1^) + *B. pumilus* treatment.

The highest increase was observed with combined Ca + Mg nanocomposite and *B. pumilus* inoculation. Further, the combined Ca + Mg nanocomposite and *B. pumilus* inoculation decreased (*p* ≤*0.05*) the TSP, TFAA, TSS, and phenolics in roots by 8%, 9%, 16%, and 14% and leaves by 24%, 15%, 16%, and 19% compared with *B. pumilus* treatment. Meanwhile, the combined Ca + Mg nanocomposite and *B. pumilus* inoculation inhibited the TSP, TFAA, TSS, and phenolics in roots by 14%, 15%, 18%, and 6% and leaves by 14%, 21%, 19%, and 2% with Cd (5 mg kg^−1^) toxicity compared with Cd (5 mg kg^−1^) + *B. pumilus* treatment. Similarly, the combined Ca + Mg nanocomposite and *B. pumilus* inoculation also decreased the TSP, TFAA, TSS, and phenolics in roots by 10%, 17%, 8%, and 7% and leaves by 11%, 12%, 10%, and 7% with Cd (10 mg kg^−1^) toxicity, compared to Cd (10 mg kg^−1^) + *B. pumilus* treatment.

### 3.5 Effect of nanocomposite (Ca + Mg) and *B. pumilus* on nutrient profile

The results showed that the soil spiking with Cd (5 mg kg^−1^, 10 mg kg^−1^) decreased the macro and micronutrients in shoots and grains of rice over the control (**Table 1**). Meanwhile, Ca + Mg nanocomposite and microbial inoculation significantly increased the macro and micronutrients under Cd (5 mg kg^−1^, 10 mg kg^−1^) toxicity. Results revealed that the Cd (5 mg kg^−1^) diminished Zn, Fe, Mg, Mn, K, and Ca in shoots by 34%, 44%, 22%, 38%, 20%, and 13% and grains by 28%, 36%, 24%, 34%, 37%, and 14% compared with control treatment. Similarly, Cd (10 mg kg^−1^) at a significant (*p* ≤*0.05*) lessened the concentration of Zn, Fe, Mg, Mn, K, and Ca in shoots by 65%, 82%, 38%, 70%, 35%, and 24%, and grains by 64%, 77%, 59%, 69%, 63%, and 24% compared with control treatment. Meanwhile, *B. pumilus* inoculation at a significant (*p* ≤*0.05*) increased the concentration of Zn, Fe, Mg, Mn, K, and Ca in shoots by 27%, 33%, 26%, 18%, 24%, and 9% and grains by 30%, 36%, 22%, 16%, 19%, and 8% as compared with control treatment. Moreover, the *B. pumilus* inoculation increased the values of Zn, Fe, Mg, Mn, K, and Ca in shoots by 60%, 88%, 42%, 57%, 38%, and 14%, and grains by 53%, 64%, 32%, 46%, 35%, and 14% with Cd (5 mg kg^−1^) contamination compared without *B. pumilus*. Under Cd (10 mg kg^−1^) contamination, *B. pumilus* inoculation at a significant (*p* ≤*0.05*) increased the concentration of Zn, Fe, Mg, Mn, K, and Ca in shoots by 146%, 358%, 41%, 158%, 37%, and 16% and grains by 152%, 217%, 82%, 115%, 60%, and 15% compared without *B. pumilus*.

**Table 1:** Macronutrients and micronutrients contents in shoots and grains under different treatments.

Treatments	Shoots						Grains					
	Micronutrients mg kg^-1^ DW			Macronutrients mg kg^-1^ DW			Micronutrients mg kg^-1^ DW			Macronutrients mg kg^-1^ DW		
	Zn	Fe	Mn	Ca	Mg	K	Zn	Fe	Mn	Ca	Mg	K
*Control*	48.67 ± 1.38d	38.90 ± 149d	50.60 ± 1.51de	231.83 ± 5.35de	2078.80 ± 61.79f	2273.83 ± 85.08f	24.10 ± 1.3de	19.60 ± 0.70d	25.53 ± 0.70c	116.17 ± 2.86de	1128.73 ± 40.27d	1241.13 ± 89.11c
*Cd (5 mg kg^-1^)*	32.07 ± 1.85f	21.97 ± 1.70f	31.50 ± 3.72g	199.97 ± 6.89f	1622.30 ± 82.72h	1812.07 ± 90.43h	17.40 ± 0.79f	12.50 ± 0.75f	16.80 ± 0.90f	99.97 ± 3.13f	865.63 ± 80.47e	777.90 ± 60.58efg
*Cd (10 mg kg^-1^)*	16.90 ± 1.51h	6.90 ± 1.51h	15.23 ± 1.62i	176.33 ± 4.37g	1290.20 ± 83.43i	1475.20 ± 59.23i	8.57 ± 0.80h	4.47 ± 0.86h	7.73 ± 0.80h	88.67 ± 2.31g	456.87 ± 55.62f	458.87 ± 45.51h
*Microbes*	61.60 ± 1.08b	51.60 ± 1.08b	59.50 ± 1.15b	251.63 ± 5.46bc	2623.23 ± 54.78bc	2824.53 ± 45.07bc	31.30 ± 0.75b	26.73 ± 0.97b	29.63 ± 0.80b	125.80 ± 2.45bc	1374.90 ± 44.40bc	1471.87 ± 46.76b
*Cd (5 mg kg^-1^) + Microbes*	51.40 ± 0.91d	41.40 ± 0.91d	49.43 ± 0.75de	227.23 ± 5.40de	2304.43 ± 61.25e	2504.43 ± 61.25e	26.60 ± 0.75cd	20.57 ± 0.96d	24.53 ± 0.60cd	114.13 ± 2.41de	1134.87 ± 41.30d	1054.10 ± 60.21d
*Cd (10 mg kg^-1^) + Microbes*	41.67 ± 1.25e	31.67 ± 1.49e	39.43 ± 1.05f	203.77 ± 3.72f	1826.23 ± 62.25g	2026.13 ± 62.25g	21.63 ± 0.80e	14.17 ± 0.75ef	16.67 ± 0.87f	101.77 ± 1.76f	830.23 ± 55.59e	735.20 ± 51.35fg
*(Ca + Mg) nanocomposite (25 mg L^-1^)*	55.77 ± 1.17c	45.77 ± 1.17c	53.90 ± 1.27cd	264.57 ± 6.03b	2734.03 ± 54.28ab	2934.03 ± 54.28ab	25.77 ± 0.45d	23.40 ± 0.98c	28.53 ± 1.12b	132.57 ± 3.39b	1516.03 ± 68.13ab	1371.53 ± 46.65bc
*Cd (5 mg kg^-1^) + (Ca + Mg) nanocomposite (25 mg L^-1^)*	41.17 ± 1.49e	31.17 ± 1.49e	39.20 ± 1.22f	241.97 ± 3.44cd	2386.30 ± 73.60de	2586.30 ± 73.60de	18.70 ± 0.95f	15.53 ± 0.70e	21.43 ± 0.65e	120.97 ± 1.51cd	1253.03 ± 82.02cd	887.97 ± 26.80ef
*Cd (10 mg kg^-1^) +(Ca + Mg) nanocomposite (25 mg L^-1^)*	25.97 ± 1.15g	15.97 ± 1.15g	23.87 ± 1.15h	219.57 ± 3.91e	1980.60 ± 84.30fg	2180.60 ± 84.30fg	13.47 ± 0.87g	8.70 ± 0.55g	13.57 ± 0.65g	109.90 ± 1.83e	867.27 ± 48.24e	667.30 ± 52.86g
*Microbes + (Ca + Mg) nanocomposite (25 mg L^-1^)*	70.57 ± 1.72a	60.57 ± 1.72a	68.57 ± 1.80a	283.90 ± 4.88a	2932.10 ± 43.93a	3132.10 ± 43.93a	35.43 ± 1.07a	32.13 ± 0.75a	35.57 ± 1.15a	141.90 ± 2.26a	1665.50 ± 64.16a	1662.10 ± 59.80a
*Cd (5 mg kg^-1^) + Microbes + (Ca + Mg) nanocomposite (25 mg L^-1^)*	60.73 ± 1.60b	50.73 ± 1.60b	58.73 ± 1.40bc	263.90 ± 5.33b	2519.53 ± 84.92cd	2719.53 ± 85.06cd	28.60 ± 0.98c	23.83 ± 0.87c	29.50 ± 0.75b	131.90 ± 2.72b	1252.87 ± 112.70cd	1231.10 ± 54.11c
*Cd (10 mg kg^-1^) + Microbes + (Ca + Mg) nanocomposite (25 mg L^-1^)*	49.37 ± 1.56d	39.37 ± 1.56d	47.10 ± 1.25e	233.60 ± 5de	2093.60 ± 55.02f	2293.57 ± 54.87f	23.07 ± 0.76e	18.47 ± 0.65d	22.43 ± 0.86de	116.60 ± 2.40de	893.23 ± 54.51e	924.80 ± 55.51de

The foliar application of Ca + Mg nanocomposite increased Zn, Fe, Mg, Mn, K, and Ca contents in shoots by 15%, 18%, 32%, 7%, 29%, and 14%, and grains by 7%, 19%, 34%, 12%, 11%, and 14% compared with control treatment. Ca + Mg nanocomposite decreased the concentration of Zn, Fe, and Mn in shoots by 19%, 25%, and 21% and increased Mg, K, and Ca 4%, 3%, and 6% with Cd (5 mg kg^−1^) contamination compared with Cd (5 mg kg^−1^) + *B. pumilus* treatment. Meanwhile, foliar application of Ca + Mg nanocomposite decreased the concentration of Zn, Fe, Mn, and K in grains by 30%, 24%, 13%, and 16% and increased Mg and Ca by 10% and 6% with Cd (5 mg kg^−1^) contamination compared with Cd (5 mg kg^−1^) + *B. pumilus* treatment. Similarly, Ca + Mg nanocomposite decreased the concentration of Zn, Fe, and Mn in shoots by 38%, 50%, and 39% and increased Mg, K, and Ca by 8%, 7%, and 8% with Cd (10 mg kg^−1^) contamination compared with Cd (10 mg kg^−1^) + *B. pumilus* treatment. Foliar application of Ca + Mg nanocomposite decreased the concentration of Zn, Fe, Mn, and K in grains by 38%, 39%, 19$, and 9% and increased Mg and Ca by 4% and 8% with Cd (10 mg kg^−1^) contamination compared with Cd (10 mg kg^−1^) + *B. pumilus* treatment.

The highest increase was observed with combined Ca + Mg nanocomposite and *B. pumilus* inoculation. Further, the combined Ca + Mg nanocomposite and *B. pumilus* inoculation at a significant (*p* ≤*0.05*) increased concentration of Zn, Fe, Mg, Mn, K, and Ca in shoots by 15%, 17%, 12%, 24%, 11% and 13% and grains by 13%, 20%, 21%, 20%, 13%, and 13% compared with *B. pumilus* treatment. Meanwhile, the combined Ca + Mg nanocomposite and *B. pumilus* inoculation increased the concentration of Zn, Fe, Mg, Mn, K, and Ca in shoots by 18%, 23%, 9%, 19%, 9%, and 16% and grains by 8%, 16%, 10%, 20%, 17%, and 16% with Cd (5 mg kg^−1^) contamination compared with Cd (5 mg kg^−1^) + *B. pumilus* treatment. Similarly, combined Ca + Mg nanocomposite and *B. pumilus* inoculation increased the concentration of Zn, Fe, Mg, Mn, K, and Ca in shoots by 19%, 24%, 15%, 19%, 13%, and 15% and grains by 7%, 30%, 8%, 35%, 26%, and 15% with Cd (10 mg kg^−1^) contamination compared with Cd (10 mg kg^−1^) + *B. pumilus* treatment.

### 3.6 Effect of nanocomposite (Ca + Mg) and *B. pumilus* on Cd uptake

**Figure 4:**
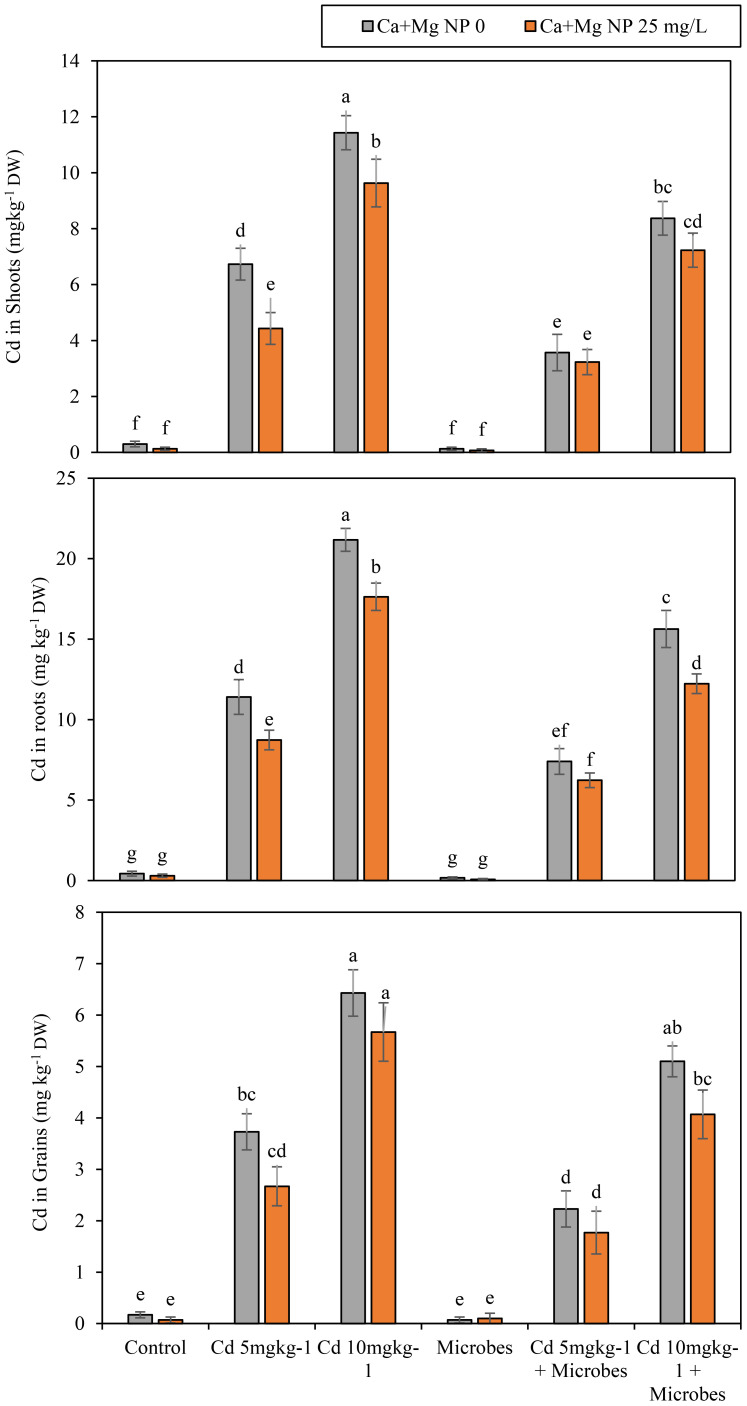
Alone and combined effect of Ca+Mg nanocomposite (25 mg L^-1^) and inoculation of *Bacillus pumilus* on Cd uptake and accumulation in roots, shoots, grains, and small letter showed the difference in significance at *p* ≤ 0.05 level with mean of three replications.

Statistically, soil spiking with Cd (5 mg kg^−1^, 10 mg kg^−1^) increased Cd levels in roots, shoots, and grains of rice over the control (**Figure 4**). Meanwhile, Ca + Mg nanocomposite and microbial inoculation significantly decreased the Cd uptake and toxicity with Cd (5 mg kg^−1^, 10 mg kg^−1^) contamination. Meanwhile, *B. pumilus* inoculation at a significant (*p* ≤*0.05*) declined the uptake of Cd in roots, shoots, and grains by 61%, 55%, and 60%, compared to control. Moreover, *B. pumilus* inoculation reduced the uptake of Cd in roots, shoots, and grains by 35%, 47%, and 40% with Cd (5 mg kg^−1^) contamination, compared to without *B. pumilus* inoculation. Under Cd (10 mg kg^−1^) contamination, *B. pumilus* inoculation at a significant (*p* ≤*0.05*) lessened the uptake of Cd in roots, shoots, and grains by 26%, 27%, and 23% compared without *B. pumilus* inoculation treatment.

Ca + Mg nanocomposite decreased the uptake of Cd in roots, shoots, and grains by 31%, 55%, and 60%, compared to the control. Meanwhile, the Ca + Mg nanocomposite augmented the uptake of Cd in roots, shoots, and grains by 18%, 24%, and 19% with Cd (5 mg kg^−1^) contamination compared with Cd (5 mg kg^−1^) + *B. pumilus* treatment. Similarly, Ca + Mg nanocomposite increased Cd uptake in roots, shoots, and grains by 13%, 15%, and 11% with Cd (10 mg kg^−1^) contamination, compared to Cd (10 mg kg^−1^) + *B. pumilus* treatment. The highest decrease was observed with combined Ca + Mg nanocomposite and *B. pumilus* inoculation. Further, the combined Ca + Mg nanocomposite and *B. pumilus* inoculation at a significant (*p* ≤*0.05*) minimized the uptake of Cd in roots, shoots, and grains by 60%, 50%, and 33% as compared with *B. pumilus* treatment. Meanwhile, the combined Ca + Mg nanocomposite and *B. pumilus* inoculation decreased the uptake of Cd in roots, shoots, and grains by 15%, 9%, and 21% with Cd (5 mg kg^−1^) contamination compared with Cd (5 mg kg^−1^) + *B. pumilus* treatment. Similarly, combined Ca + Mg nanocomposite and *B. pumilus* inoculation reduced the uptake of Cd in roots, shoots, and grains by 22%, 13%, and 20% with Cd (10 mg kg^−1^) contamination compared with Cd (10 mg kg^−1^) + *B. pumilus* treatment.

## 4 Discussion

Several studies showed that the application of exogenic CaO and MgO NPs alleviates Cd toxicity in different crops (Li et al., 2022; Nazir et al., 2022). Similarly, *B. pumilus* strains increased plant growth by decreasing Cd contamination in soil (Shahzad et al., 2021; Maslennikova et al., 2023). However, there is a gap in research regarding the combined effect of Ca–Mg nanocomposite and microbes on plants and their mitigation mechanism. Therefore, the present study examined the combined effect of Ca–Mg and *B. pumilus* inoculation on physiology, photosynthetic pigments, oxidative stress, Cd uptake, and accumulation in rice.

**Figure 5:**
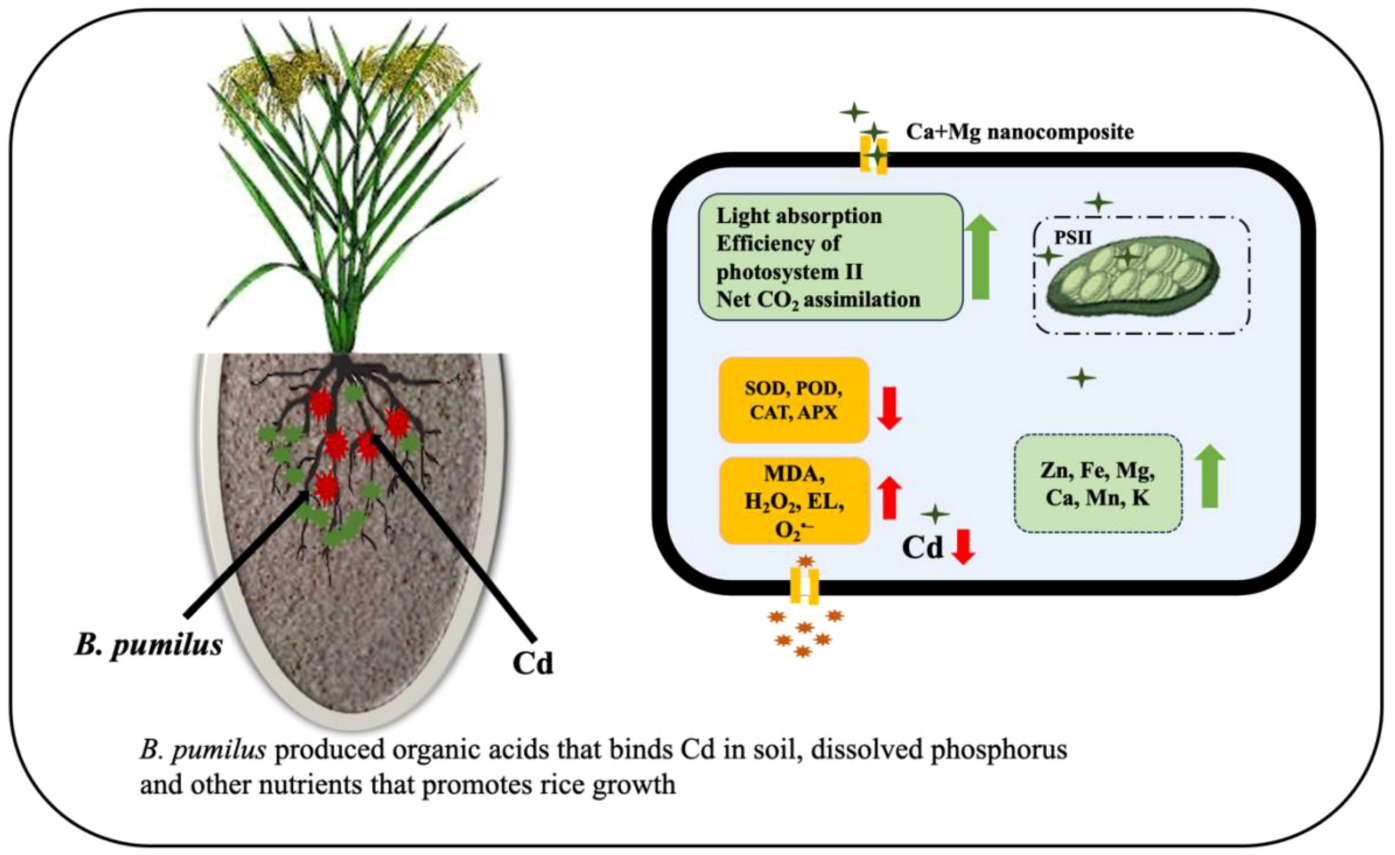
Proposed mechanism of Ca + Mg nanocomposite and *B*. *pumilus* to mitigate Cd toxicity and enhance rice growth and yield.

After Cd uptake, the high excitation energy of thylakoid situated photosynthetic electron transport was produced, which ultimately promotes ROS synthesis (MDA, H_2_O_2,_ and EL) (Huihui et al., 2020). Cd also reduced the production of SOD, POD, CAT, and APX, which may hinder excessive scavenging of oxidative stress species. MgO NPs enhanced light absorption, photosynthetic function, photosystem II (PSII) efficacy, Fv/Fm, and the effective quantum yield of PSII photochemistry (FPSII). Magnesium also benefits net CO_2_ absorption in several plant species and mitigates heavy metal stress (Tränkner et al., 2016; Samborska et al., 2018; Faizan et al., 2021). To minimize the toxic effects in heavy-metal-stressed plants, usually caused by the generation of reactive oxygen species (ROS), antioxidative protective mechanisms are activated, including the antioxidant enzymes CAT, POD, and SOD (Ahmad et al., 2018). Similarly, CaO NPs enhanced growth, antioxidative enzymes, and nutrient profile by inhibiting Cd uptake and toxicity in barley seedlings (Nazir et al., 2022). *B. pumilus* produces organic acids that bind C and solubilize phosphorus and other nutrients, which help plant growth (Sharma et al., 2013). The proposed mechanism of the Ca + Mg nanocomposite and *B. pumilus* strain is shown in **Figure 5**. Roots are exposed directly to the soil, and the Casparian strip might be an effective barrier to decrease Cd uptake and translocation due to the sole structure of the endothelial layer (Wu et al., 2018; Guo et al., 2021). Earlier findings discovered that Cd content in rice roots was 10 times higher than in upground plant parts, and a major portion of Cd accumulated in the cell wall, demonstrating that the root cell wall efficiently reduced Cd translocation to shoots and leaves (Liu et al., 2016; Yu et al., 2020). Cd concentration was significantly minimized with Ca, signifying an intervention between Ca^2+^ and Cd^2+^ ions. Cd is a nonessential and toxic element for rice plants, and Ca transporters enter the cells due to the chemical similarity (Tian et al., 2016; Ye et al., 2020). Through specific translocation channels, Ca^2+^ ions impede the uptake and translocation of Cd^2+^ ions by the roots, subsequently reducing the Cd concentration in plant parts (Kanu et al., 2019). Ca contents in the cytoplasm increased significantly, and signals were conveyed quickly among cells, allowing plants to mitigate Cd toxicity (Guo et al., 2018). Cd toxicity increased rice’s amino groups, hydroxyl groups, cellulose, epoxide, and reactive oxygen species richness, causing structural damage to the plasma membrane and cell wall. At the same time, Ca minimized these unfavorable effects (Ye et al., 2020). Previous analyses indicated that Cd toxicity caused structural, chlorophyll alterations, primarily by replacing Mg^2+^ ions, leading to the breakdown of chlorophyll fragments (Kanu et al., 2019). Similarly, Mg concentrations correlate negatively with Cd concentrations in rice leaves and shoots (Khaliq et al., 2019).

Cadmium is a non-essential element for plant growth with no biological function and inhibits the biomass of various plant species, including root and shoot length, dry weight, and SPAD values (Jia et al., 2020; Yang et al., 2020; Li et al., 2021). Similar results were found in the current study: Cd toxicity decreased rice plant growth parameters, including root and shoot length, root and shoot fresh and dry weight, number of tillers, and spike length, as shown in **Table 2**. Several studies have demonstrated that the exogenous application of NPs can alleviate Cd toxicity in wheat and rice, including Fe, Cu, TiO_2_, and ZnO NPs (Hussain et al., 2018; Noman et al., 2020; Irshad et al., 2021; Manzoor et al., 2021). It has been reported that CaO NPs could promote root development, growth rate, and seed yield, and supply Ca in soybean and peanut (Liu and Lal, 2014, 2015). Ca and Mg are beneficial elements and enhance the plant growth. A previous study reported that the application of Ca and Mg improved growth by reducing Cd accumulation and translocation in rice (Li et al., 2022). Similarly, Mg input to chloroplast significantly enhanced the photosynthesis in rice (Li et al., 2020). Several microbes are well-known for detoxifying, transferring, and accumulating heavy metals (Pathania and Srivastava, 2020). A previous study reported that seed inoculation with *B. pumilus* increased maize growth by decreasing Cd toxicity (Shahzad et al., 2021). Fatemi et al. (2020) reported that the combined application of lead (Pb)-resistant microbes and silicon NPs improved the growth of coriander (*Coriandrum sativum* L.) under Pb stress. Previous studies showed that applying silica NPs with two microbial strains (*Azotobacter chroococcum* and *Pseudomonas koreensis*) improved the physiology and SPAD values of barely under saline stress (Alharbi et al., 2022). Our study also showed similar results: the combined application of Ca–Mg nanocomposite and *B. pumilus* inoculation minimizes Cd toxicity and increases plant growth factors (**Table 3**).

**Table 2:** Growth parameters under different treatments.

Treatments									
	RL	SL	RFW	SFW	RDW	SDW	NoT	NoG	SpL
*Control*	10 ± 0.41cd	70 ± 5.21cd	15 ± .56b	103 ± 1.80cd	2 ± 0.06bc	40 ± 1.74cd	10 ± 0.77bcd	30 ± 0.66d	12 ± 1.13cd
*Cd (5 mg kg^-1^)*	8 ± 0.36f	49 ± 2.95fg	12 ± 0.75cde	89 ± 2.38ef	1 ± 0.03ef	33 ± 1.64ef	6 ± 0.77ef	24 ± 1.31e	10 ± 1.02def
*Cd (10 mg kg^-1^)*	6 ± 0.44h	38 ± 5.05g	9 ± 0.61e	70 ± 4.27g	1 ± 0.04g	25 ± 1.64g	4 ± 1.18f	19 ± 0.89f	7 ± 1.26f
*Microbes*	13 ± 0.36b	85 ± 2.63ab	19 ± 1.69a	113 ± 2.65b	2 ± 0.078a	47 ± 1.72ab	12 ± 1.18ab	34 ± 0.86bc	14 ± 0.75bc
*Cd (5 mg kg^-1^) + Microbes*	9 ± 0.41ef	54 ± 2.70ef	13 ± 0.92bcd	98 ± 1.81cd	1 ± 0.12cde	37 ± 2.30cdef	9 ± 1.18bcde	28 ± 1.31d	11 ± 0.76cde
*Cd (10 mg kg^-1^) + Microbes*	6 ± 0.73gh	45 ± 3.72fg	10 ± 0.88de	84 ± 3.35f	1 ± 0.04fg	32 ± 2.12f	7 ± 1.18def	24 ± 1.08e	9 ± 1.09ef
*(Ca + Mg) nanocomposite (25 mg L^-1^)*	13 ± 0.61b	79 ± 3.13bc	18 ± 0.55a	113 ± 3.98b	2 ± 0.06a	46 ± 2.35ab	12 ± 1.18abc	36 ± 1.08b	16 ± 0.83ab
*Cd (5 mg kg^-1^) + (Ca + Mg) nanocomposite (25 mg L^-1^)*	10 ± 0.35cde	63 ± 2.30de	14 ± 1.17bc	98 ± 3.16cd	1 ± 0.03bcd	38 ± 1.56cde	9 ± 1.18cde	29 ± 0.43d	12 ± 1.10cde
*Cd (10 mg kg^-1^) +(Ca + Mg) nanocomposite (25 mg L^-1^)*	8 ± 0.32fg	53 ± 4.21ef	11 ± 0.35cde	82 ± 3.27f	1 ± 0.04def	33 ± 1.60ef	7 ± 1.18ef	24 ± 0.43e	10 ± 0.60ef
*Microbes + (Ca + Mg) nanocomposite (25 mg L^-1^)*	15 ± 0.76a	92 ± 4.93a	21 ± 1.42a	124 ± 3.21a	2 ± 0.08a	51 ± 1.93a	15 ± 0.77a	39 ± 0.66a	17 ± 0.88a
*Cd (5 mg kg^-1^) + Microbes + (Ca + Mg) nanocomposite (25 mg L^-1^)*	11 ± 0.80bc	70 ± 3.58cd	15 ± 1.16b	107 ± 1.23bc	2 ± 0.07b	42 ± 2.74bc	10 ± 1.18bc	33 ± 0.86c	14 ± 0.83bc
*Cd (10 mg kg^-1^) + Microbes + (Ca + Mg) nanocomposite (25 mg L^-1^)*	9 ± 0.61def	60 ± 3.18de	12 ± 1.15bcd	97 ± 3.20de	1 ± 0.10de	36 ± 2.02f	9 ± 1.18bcde	29 ± 1.31d	12 ± 1.20cde

**Table 3:** Photosynthetic and gas exchange parameters under different treatments.

Treatments									
	SPAD	Chl a	Chl b	Total chl	Carotenoids	Pn	Tr	WUE	SC
*Control*	40 ± 2.40cde	3 ± 0.20bcd	2 ± 0.06bcd	5 ± 0.25bcd	1.2 ± 0.10bc	10 ± 0.30bc	3 ± 0.15bc	7 ± 0.30cd	2 ± 0.12cd
*Cd (5 mg kg^-1^)*	35 ± 3.09def	2 ± 0.20def	1 ± 0.10f	4 ± 0.25efg	0.8 ± 0.10def	8 ± 0.40de	2 ± 0.20cde	5 ± 0.15fg	1 ± 0.14ef
*Cd (10 mg kg^-1^)*	26 ± 3.03g	2 ± 0.25f	1 ± 0.10g	3 ± 0.20g	0.5 ± 0.05f	6 ± 0.50f	2 ± 0.20e	4 ± 0.40h	1 ± 0.10f
*Microbes*	49 ± 2.94ab	3 ± 0.30ab	2 ± 0.10b	6 ± 0.46b	1.4 ± 0.10ab	12 ± 0.60b	3 ± 0.20a	8 ± 0.40ab	2 ± 0.14ab
*Cd (5 mg kg^-1^) + Microbes*	38 ± 2.25cdef	3 ± 0.31cde	2 ± 0.10de	4 ± 0.31cde	1 ± 0.10cd	10 ± 0.50c	2 ± 0.15bcd	6 ± 0.60def	2 ± 0.14de
*Cd (10 mg kg^-1^) + Microbes*	31 ± 2.59fg	2 ± 0.20ef	1 ± 0.10f	4 ± 0.31fg	0.8 ± 0.10def	7 ± 0.60e	2 ± 0.20de	5 ± 0.35g	1 ± 0.07ef
*(Ca + Mg) nanocomposite (25 mg L^-1^)*	44 ± 3.13bc	3 ± 0.20abc	2 ± 0.10bc	5 ± 0.35bc	1.3 ± 0.10ab	11 ± 0.40b	3 ± 0.20ab	7 ± 0.30bc	2 ± 0.12bc
*Cd (5 mg kg^-1^) + (Ca + Mg) nanocomposite (25 mg L^-1^)*	40 ± 2.39cde	3 ± 0.26bcde	2 ± 0.06de	5 ± 0.32def	0.9 ± 0.10de	10 ± 0.40cd	3 ± 0.20bcd	6 ± 0.35cde	2 ± 0.09cd
*Cd (10 mg kg^-1^) +(Ca + Mg) nanocomposite (25 mg L^-1^)*	34 ± 2.51efg	2 ± 0.25def	1 ± 0.10ef	4 ± 0.31efg	0.7 ± 0.10ef	8 ± 0.45e	2 ± 0.25de	5 ± 0.30efg	1 ± 0.0.08ef
*Microbes + (Ca + Mg) nanocomposite (25 mg L^-1^)*	54 ± 2.55a	4 ± 0.20a	2 ± 0.10a	7 ± 0.30a	1.5 ± 0.10a	14 ± 0.50a	4 ± 0.26a	9 ± 0.50a	3 ± 0.14a
*Cd (5 mg kg^-1^) + Microbes + (Ca + Mg) nanocomposite (25 mg L^-1^)*	43 ± 1.75bcd	3 ± 0.25bcd	2 ± 0.15bcd	5 ± 0.53bcd	1.2 ± 0.10bc	11 ± 0.31b	3 ± 0.35bc	7 ± 0.30bcd	2 ± 0.19bcd
*Cd (10 mg kg^-1^) + Microbes + (Ca + Mg) nanocomposite (25 mg L^-1^)*	36 ± 2.01def	2 ± 0.15def	1 ± 0.15cd	4 ± 0.31def	0.9 ± 0.05cde	9 ± 0.35cd	2 ± 0.20cde	6 ± 0.30defg	2 ± 0.19de

The literature confirmed that photosynthetic pigments are considered fundamental markers of heavy metals-induced oxidative stress (Rizwan et al., 2018). Previous studies demonstrated that CaO NPs increased chlorophyll content and gas exchange characteristics by reducing arsenic toxicity in barley. Ilyas et al. (2022) stated that seed inoculation with *Bacillus* sp. strains increased the chlorophyll contents in wheat plants. Moreover, chlorophyll content and gas exchange attributes significantly increased with the combined application of Ca + Mg and microbial inoculation. Previous studies also agreed that the combined application of ZnO NPs and Cr-resistant microbes increased chlorophyll content and gas exchange characteristics in wheat (Ahmad et al., 2022). The present study demonstrated similar findings: Ca + Mg enhanced chlorophyll and gas exchange characteristics in rice leaves under Cd contamination (**Table 3**).

Antioxidant enzymes activity was reduced with increasing Cd concentration (Shi et al., 2020). Cd toxicity inhibits growth and triggers oxidative damage due to the release of ROS (Marques et al., 2019). ROS damage biomolecules, proteins, carbohydrates, and lipids, particularly in membranes, harming the integral membrane and ultimately leading to cell death (Dar et al., 2017). Researchers found that Cd contamination increased MDA, H_2_O_2,_ EL, and O_2_^•–^ due to ROS generation (Du et al., 2020; Kaya et al., 2020; Ali et al., 2022a). Similar outcomes were found in our study: Cd contamination decreased antioxidant enzyme activity and increased oxidative damage by producing ROS. Similarly, the combined application of *Staphylococcus aureus* and ZnO NPs reduced ROS production and increased antioxidant enzyme activities in wheat under Cr-contaminated soil (Ahmad et al., 2022). Our study showed similar results: the combined application of Ca–Mg and *B. pumilus* inoculation decreased ROS production by reducing EL%, MDA, H_2_O_2_, and O_2_^•–^ concentrations and increased antioxidant enzyme activities by increasing POD, SOD, APX, and CAT concentrations in roots and leaves (**Figures 2A, B**).

Cd toxicity increased the osmolytes (proline and glycine betaine), sugar content, and secondary metabolites in mustard (*Brassica juncea*) plants (Ahmad et al., 2016). In contrast, the application of *Pseudomonas aeruginosa and Burkholderia gladioli*) decreased Cd toxicity in *Solanum lycopersicum* seedlings by modulating the expression of secondary metabolites (Khanna et al., 2019). Similar outcomes were found in our study: *B. pumilus* inoculation decreased Cd toxicity by regulating secondary metabolites. The current study showed that Cd toxicity significantly increased secondary metabolites such as TSP, TFAA, TSS, and phenolics in roots and shoots, while combined application of Ca–Mg and *B. pumilus* inoculation slightly decreased secondary metabolism in leaves and roots (**Figures 3A, B**).

Nano-fertilization has been considered a game-changer in addressing nutritional insecurity, especially in developing countries (Wang et al., 2020; Cao et al., 2022);. The application of CaO NPs decreased the Cd concentration in the roots, shoots, and grains of barley plants and increased the macro and micronutrients (Nazir et al., 2022). Similarly, gallic acid increased wheat growth and decreased the uptake of Cr in roots, shoots and grains under tannery wastewater stress (Nafees et al., 2024b). Previously, Ca and Mg reduced the Cd concentration in rice roots, shoots, and grains (Li et al., 2022). Similar results were found in our study: the combined application of foliar Ca–Mg and *B. pumilus* decreased the Cd concentration in rice roots, shoots, and grains (**Figure 4**) and increased the macro and micronutrients (Zn, Fe, Mg, Mn, K, and Ca) in shoots and grains (**Table 1**). Therefore, during phytoremediation, the foliar application of Ca + Mg nanocomposite and microbial (*B. pumilus*) inoculation will not only raise the market value of agricultural products but also improve the nutritional value of rice grains, which is of great significance in addressing hidden hunger.

## 5 Conclusion

The present study investigated how cadmium toxicity reduced plant growth, biomass, and gas exchange characteristics in rice. Additionally, Cd-stressed rice demonstrated increased MDA, H_2_O_2_, EL, and O_2_^•–^ levels and higher Cd concentration. However, the application of Ca + Mg nanocomposite and *B. pumilus* individually modulated the antioxidant system of treated rice by improving the activity of CAT, SOD POD, and APX. Moreover, the combined application of Ca + Mg nanocomposite and *B. pumilus* further enhanced the growth and physiochemical features of rice seedlings grown under both standard and Cd-contaminated conditions. Further studies are required to understand the molecular mechanisms involved in Cd tolerance in different crops through the synergistic application of Ca + Mg nanocomposite and *B. pumilus*.

## Data availability statement

The raw data supporting the conclusions of this article will be made available by the authors, without undue reservation.

## Author contributions

MuA: Writing – review & editing, Writing – original draft, Resources, Methodology, Formal analysis, Conceptualization. MN: Writing – review & editing, Resources, Data curation, Conceptualization. MW: Conceptualization, Resources, Writing – review & editing, Data curation. SOA: Data curation, Writing – review & editing, Formal analysis. KA-G: Investigation, Formal analysis, Writing – review & editing. MoA: Methodology, Writing – review & editing. HZ: Software, Formal analysis, Writing – review & editing. SA: Writing – review & editing, Supervision, Resources, Project administration, Funding acquisition. FL: Supervision, Resources, Project administration, Funding acquisition, Writing – review & editing.

## References

[B1] Abbas T. Rizwan M. Ali S. Zia-ur-Rehman M. Qayyum M. F. Abbas F.. (2017). Effect of biochar on cadmium bioavailability and uptake in wheat (Triticum aestivum L.) grown in a soil with aged contamination. Ecotoxicology Environ. Saf. 140, 37–47. 10.1016/j.ecoenv.2017.02.028 28231504

[B2] Aebi H. (1984). Catalase in *vitro* Methods in enzymology Vol. 105 (Netherlands: Academic Press, Elsevier), 121–126. doi: 10.1016/s0076-6879(84)05016-3 6727660

[B3] Ahmad P. Abd Allah E. F. Hashem A. Sarwat M. Gucel S. (2016). Exogenous application of selenium mitigates cadmium toxicity in Brassica juncea L.(Czern & Cross) by up-regulating antioxidative system and secondary metabolites. J. Plant Growth Regul. 35, 936–950. doi: 10.1007/s00344-016-9592-3

[B4] Ahmad P. Ahanger M. A. AlYemeni M. N. Wijaya L. Alam P. (2018). Exogenous application of nitric oxide modulates osmolyte metabolism, antioxidants, enzymes of ascorbate-glutathione cycle and promotes growth under cadmium stress in tomato. Protoplasma 255, 79–93. doi: 10.1007/s00709-017-1132-x 28643085

[B5] Ahmad S. Mfarrej M. F. B. El-Esawi M. A. Waseem M. Alatawi A. Nafees M.. (2022). Chromium-resistant Staphylococcus aureus alleviates chromium toxicity by developing synergistic relationships with zinc oxide nanoparticles in wheat. Ecotoxicology Environ. Saf. 230, 113142. doi: 10.1016/j.ecoenv.2021.113142 34990991

[B6] Alengebawy A. Abdelkhalek S. T. Qureshi S. R. Wang M. Q. (2021). Heavy metals and pesticides toxicity in agricultural soil and plants: ecological risks and human health implications. Toxics 9 (3), 42. doi: 10.3390/toxics9030042 33668829 PMC7996329

[B7] Alharbi K. Rashwan E. Mohamed H. H. Awadalla A. Omara A. E.-D. Hafez E. M.. (2022). Application of silica nanoparticles in combination with two bacterial strains improves the growth, antioxidant capacity and production of barley irrigated with saline water in salt-affected soil. Plants 11, 2026. doi: 10.3390/plants11152026 35956503 PMC9370161

[B8] Ali S. Mfarrej M. F. B. Rizwan M. Hussain A. Akram N. A. Rizwan M.. (2022a). Zinc fortification and alleviation of cadmium stress by application of lysine chelated zinc on different varieties of wheat and rice in cadmium stressed soil. Chemosphere 295, 133829. doi: 10.1016/j.chemosphere.2022.133829 35120959

[B9] Ali S. Mfarrej M. F. B. Rizwan M. Hussain A. Shahid M. J. Wang X.. (2022b). Microbe-citric acid assisted phytoremediation of chromium by castor bean (*Ricinus communis* L.). Chemosphere. 296, 134065, 0045–6535. doi: 10.1016/j.chemosphere.2022.134065 35202665

[B10] Ali S. Rizwan M. Noureen S. Anwar S. Ali B. Naveed M.. (2019). Combined use of biochar and zinc oxide nanoparticle foliar spray improved the plant growth and decreased the cadmium accumulation in rice (*Oryza sativa* L.) plant. Environ. Sci. pollut. Res. 26, 11288–11299. doi: 10.1007/s11356-019-04554-y 30793248

[B11] Amacher M. C. (1996). Nickel, cadmium, and lead. Methods of Soil Analysis: Part 3. Chem. Methods 5, 739–768. doi: 10.2136/sssabookser5.3.c28

[B12] ASTDR (2021). Substance Priority List=ASTDR. Available online at: https://www.atsdr.cdc.gov/spl/ (Accessed 11 November 2021).

[B13] Ayyaz A. Fang R. Ma J. Hannan F. Huang Q. Sun Y.. (2022). Calcium nanoparticles (Ca-NPs) improve drought stress tolerance in Brassica napus by modulating the photosystem II, nutrient acquisition and antioxidant performance. NanoImpact 28, 100423. doi: 10.1016/j.impact.2022.100423 36084849

[B14] Babu S. Singh R. Yadav D. Rathore S. S. Raj R. Avasthe R.. (2022). Nanofertilizers for agricultural and environmental sustainability. Chemosphere 292, 133451. doi: 10.1016/j.chemosphere.2021.133451 34973251

[B15] Bhardwaj A. K. Arya G. Kumar R. Hamed L. Pirasteh-Anosheh H. Jasrotia P.. (2022). Switching to nanonutrients for sustaining agroecosystems and environment: the challenges and benefits in moving up from ionic to particle feeding. J. Nanobiotechnol. 20, 1–28. doi: 10.1186/s12951-021-01177-9 PMC8728941 34983548

[B16] Bouyoucos G. J. (1962). Hydrometer method improved for making particle size analyses of soils. Agron. J. 54, 464–465. doi: 10.2134/agronj1962.00021962005400050028x

[B17] Bradford M. M. (1976). A rapid and sensitive method for the quantitation of microgram quantities of protein utilizing the principle of protein-dye binding. Analyt. Biochem. 72, 248–254. doi: 10.1016/0003-2697(76)90527-3 942051

[B18] Cao X. Yue L. Wang C. Luo X. Zhang C. Zhao X.. (2022). Foliar application with iron oxide nanomaterials stimulate nitrogen fixation, yield, and nutritional quality of soybean. ACS Nano 16, 1170–1181. doi: 10.1021/acsnano.1c08977 35023717

[B19] Chen W. Li H. (2018). Cost-effectiveness analysis for soil heavy metal contamination treatments. Water Air Soil pollut. 229, 1–13. doi: 10.1007/s11270-018-3784-3

[B20] Dar M. I. Naikoo M. I. Khan F. A. Rehman F. Green I. D. Naushin F.. (2017). “An introduction to reactive oxygen species metabolism under changing climate in plants,” in Reactive Oxygen Species and Antioxidant Systems in Plants: Role and Regulation under Abiotic Stress (Springer, Singapore), 25–52.

[B21] Das A. Mandal A. C. Roy S. Nambissan P. M. G. (2018). Internal defect structure of calcium doped magnesium oxide nanoparticles studied by positron annihilation spectroscopy. AIP Adv. 8, 095013. doi: 10.1063/1.5001105

[B22] Dionisio-Sese M. L. Tobita S. (1998). Antioxidant responses of rice seedlings to salinity stress. Plant Sci. 135, 1–9. doi: 10.1016/S0168-9452(98)00025-9

[B23] Du B. Zhou J. Lu B. Zhang C. Li D. Zhou J.. (2020). Environmental and human health risks from cadmium exposure near an active lead, zinc mine and a copper smelter. China. Sci. Total Environ. 720, 137–585. doi: 10.1016/j.scitotenv.2020.137585 32135280

[B24] Faizan M. Faraz A. Mir A. R. Hayat S. (2021). Role of zinc oxide nanoparticles in countering negative effects generated by cadmium in *lycopersicon esculentum* . J. Plant Growth Regul. 40, 101–115. doi: 10.1007/s00344-019-10059-2

[B25] FAO F. (2017). Food and Agriculture Organization of the United Nations Statistics Division (FAOSTAT).

[B26] Fatemi H. Pour B. E. Rizwan M. (2020). Isolation and characterization of lead (Pb) resistant microbes and their combined use with silicon nanoparticles improved the growth, photosynthesis and antioxidant capacity of coriander (*Coriandrum sativum* L.) under Pb stress. Environ. pollut. 266, 114982. doi: 10.1016/j.envpol.2020.114982 32650299

[B27] Guo Y. Mao K. Cao H. Ali W. Lei D. Teng D.. (2021). Exogenous selenium (cadmium) inhibits the absorption and transportation of cadmium (selenium) in rice. Environ. pollut. 268, 115829. doi: 10.1016/j.envpol.2020.115829 33160738

[B28] Guo J. Zhou R. Ren X. Jia H. Hua L. Xu H.. (2018). Effects of salicylic acid, Epi-brassinolide and calcium on stress alleviation and Cd accumulation in tomato plants. Ecotoxicol. Environ. Safety. 157, 491–496. doi: 10.1016/j.ecoenv.2018.04.010 29685680

[B29] Hamilton P. B. Van Slyke D. D. Lemish S. (1943). The gasometric determination of free amino acids in blood filtrates by the ninhydrin-carbon dioxide method. Journal of Biological. Chemistry 150, 231–250. doi: 10.1016/S0021-9258(18)51268-0

[B30] Hassan Z.u. Ali S. Rizwan M. Ibrahim M. Nafees M. Waseem M. (2017). “Role of Bioremediation Agents (Bacteria, Fungi, and Algae) in Alleviating Heavy Metal Toxicity,” in Probiotics in Agroecosystem. Eds. Kumar V. Kumar M. Sharma S. Prasad R. (Springer, Singapore). doi: 10.1007/978-981-10-4059-7_27

[B31] Hassanpouraghdam M. B. Mehrabani L. V. Tzortzakis N. (2020). Foliar application of nano-zinc and iron affects physiological attributes of Rosmarinus officinalis and quietens NaCl salinity depression. J. Soil Sci. Plant Nutri. 20, 335–345. doi: 10.1007/s42729-019-00111-1

[B32] Hong J. Wang L. Sun Y. Zhao L. Niu G. Tan W.. (2016). Foliar applied nanoscale and microscale CeO2 and CuO alter cucumber (Cucumis sativus) fruit quality. Sci.Total Environ. 563, 904–911. doi: 10.1016/j.scitotenv.2015.08.029 26351199

[B33] Hou D. O’Connor D. Igalavithana A. D. Alessi D. S. Luo J. Tsang D. C.. (2020). Metal contamination and bioremediation of agricultural soils for food safety and sustainability. Nat. Rev. Earth Environ. 1, 366–381. doi: 10.1038/s43017-020-0061-y

[B34] Huihui Z. Xin L. Zisong X. Yue W. Zhiyuan T. Meijun A.. (2020). Toxic effects of heavy metals pb and cd on mulberry (Morus alba L.) seedling leaves: photosynthetic function and reactive oxygen species (ROS) metabolism responses. Ecotoxicol. Environ. Saf. 195, 110469. doi: 10.1016/j.ecoenv.2020.110469 32179235

[B35] Hussain A. Ali S. Rizwan M. ur Rehman M. Z. Javed M. R. Imran M.. (2018). Zinc oxide nanoparticles alter the wheat physiological response and reduce the cadmium uptake by plants. Environ. pollut. 242, 1518–1526. doi: 10.1016/j.envpol.2018.08.036 30144725

[B36] Ilyas N. Akhtar N. Yasmin H. Sahreen S. Hasnain Z. Kaushik P.. (2022). Efficacy of citric acid chelate and Bacillus sp. in amelioration of cadmium and chromium toxicity in wheat. Chemosphere 290, 133342. doi: 10.1016/j.chemosphere.2021.133342 34922965

[B37] Irshad M. A. Nawaz R. ur Rehman M. Z. Adrees M. Rizwan M. Ali S.. (2021). Synthesis, characterization and advanced sustainable applications of titanium dioxide nanoparticles: A review. Ecotoxicol. Environ. Safety. 212, 111978. doi: 10.1016/j.ecoenv.2021.111978 33561774

[B38] Ishfaq M. Yanting Z. Yongqi W. Xuexian L. (2021). Magnesium limitation leads to transcriptional down-tuning of auxin synthesis, transport, and signaling in the tomato root. Front. Plant Sci. 12. doi: 10.3389/fpls.2021.802399 PMC8733655 35003191

[B39] Ishfaq M. Yongqi W. Minwen Y. Zheng Z. Liangquan W. Chunjian L.. (2022). Physiological essence of magnesium in plants and its widespread deficiency in the farming system of China. Front. Plant Sci. 13. doi: 10.3389/fpls.2022.802274 PMC9085447 35548291

[B40] Jackson M. (1962). Interlayering of expansible layer silicates in soils by chemical weathering. Clays Clay Mineral. 11, 29–46. doi: 10.1346/CCMN.1962.0110104

[B41] Jana S. Choudhuri M. A. (1982). Senescence in submerged aquatic angiosperms: effects of heavy metals. New Phytol. 90, 477–484. doi: 10.1111/j.1469-8137.1982.tb04480.x

[B42] Jia H. Hou D. O’Connor D. Pan S. Zhu J. Bolan N. S.. (2020). Exogenous phosphorus treatment facilitates chelation-mediated cadmium detoxification in perennial ryegrass (Lolium perenne L.). J. Hazardous Materials 389, 121849. 10.1016/j.jhazmat.2019.121849 31843404

[B43] Kanu A. S. Ashraf U. Mo Z. Sabir S. U. R. Baggie I. Charley C. S.. (2019). Calcium amendment improved the performance of fragrant rice and reduced metal uptake under cadmium toxicity. Environ. Sci. pollut. Res. 26, 24748–24757. doi: 10.1007/s11356-019-05779-7 31240656

[B44] Kaya C. Ashraf M. AlYemeni M. N. Corpas F. J. Ahmad P. (2020). Salicylic acid-induced nitric oxide enhances arsenic toxicity tolerance in maize plants by upregulating the ascorbate-glutathione cycle and glyoxalase system. J. Hazard. Mater. 399, 123020. doi: 10.1016/j.jhazmat.2020.123020 32526442

[B45] Khalid F. Asif K. Rasheed Y. Ashraf H. Maqsood M. F. Rana S.. (2023). Nano priming for boosting growth and resilience in crops under abiotic stresses. Biocatal. Agric. Biotechnol. 53, 102892. doi: 10.1016/j.bcab.2023.102892

[B46] Khaliq M. A. James B. Chen Y. H. Saqib H. S. A. Li H. H. Jayasuriya P.. (2019). Uptake, translocation, and accumulation of Cd and its interaction with mineral nutrients (Fe, Zn, Ni, Ca, Mg) in upland rice. Chemosphere 215, 916–924. doi: 10.1016/j.chemosphere.2018.10.077 30408887

[B47] Khanna K. Jamwal V. L. Sharma A. Gandhi S. G. Ohri P. Bhardwaj R.. (2019). Supplementation with plant growth promoting rhizobacteria (PGPR) alleviates cadmium toxicity in Solanum lycopersicum by modulating the expression of secondary metabolites. Chemosphere 230, 628–639. doi: 10.1016/j.chemosphere.2019.05.072 31128509

[B48] Li J. Ma Y. Xie Y. (2021). Stimulatory effect of Fe3O4 nanoparticles on the growth and yield of pseudostellaria heterophylla *via* improved photosynthetic performance. HortScience 56 (7), 753–761.

[B49] Li X. Teng L. Fu T. He T. Wu P. (2022). Comparing the effects of calcium and magnesium ions on accumulation and translocation of cadmium in rice. Environ. Sci. pollut. Res. 29, 41628–41639. doi: 10.1007/s11356-021-17923-3 35094265

[B50] Li J. Yokosho K. Liu S. Cao H. R. Yamaji N. Zhu X. G.. (2020). Diel magnesium fluctuations in chloroplasts contribute to photosynthesis in rice. Nat. Plants 6, 848–859. doi: 10.1038/s41477-020-0686-3 32541951

[B51] Liu Z. Zhang Q. Han T. Ding Y. Sun J. Wang F.. (2016). Heavy metal pollution in a soil-rice system in the yangtze river region of china. Int. J. Environ. Res. Public Health 13 (1), 63. 10.3390/ijerph13010063 PMC4730454 26703698

[B52] Lichtenthaler H. K. (1987). Chlorophylls and carotenoids: pigments of photosynthetic biomembranes Methods in enzymology Vol. 148 (Netherlands: Academic Press, Elsevier), 350–382. doi: 10.1016/0076-6879(87)48036-1

[B53] Liu R. Lal R. (2014). Synthetic apatite nanoparticles as a phosphorus fertilizer for soybean (*Glycine max*). Scientic Rep. 4, 5686. doi: 10.1038/srep05686 PMC5375976 25023201

[B54] Liu R. Lal R. (2015). Potentials of engineered nanoparticles as fertilizers for increasing agronomic productions. Sci. Total Environ. 514, 131–139. doi: 10.1016/j.scitotenv.2015.01.104 25659311

[B55] Liu J. Li N. Zhang W. Wei X. Tsang D. C. Sun Y.. (2019). Thallium contamination in farmlands and common vegetables in a pyrite mining city and potential health risks. Environ. pollut. 248, 906–915. doi: 10.1016/j.envpol.2019.02.092 30856506

[B56] Liu W. Zhou Q. An J. Sun Y. Liu R. (2010). Variations in cadmium accumulation among Chinese cabbage cultivars and screening for Cd-safe cultivars. J. Hazard. Mater. 173, 737–743. doi: 10.1016/j.jhazmat.2009.08.147 19775811

[B57] Lwalaba J. L. W. Louis L. T. Zvobgo G. Richmond M. E. A. Fu L. Naz S.. (2020). Physiological and molecular mechanisms of cobalt and copper interaction in causing phyto-toxicity to two barley genotypes differing in Co tolerance. Ecotoxicol. Environ. Safety. 187, 109866. doi: 10.1016/j.ecoenv.2019.109866 31677568

[B58] Manzoor N. Ahmed T. Noman M. Shahid M. Nazir M. M. Ali L.. (2021). Iron oxide nanoparticles ameliorated the cadmium and salinity stresses in wheat plants, facilitating photosynthetic pigments and restricting cadmium uptake. Sci. Total Environ. 769, 145221. doi: 10.1016/j.scitotenv.2021.145221 33736258

[B59] Maqsood M. F. Shahbaz M. Khalid F. Rasheed Y. Asif K. Naz N.. (2023). Biogenic nanoparticles application in agriculture for ROS mitigation and abiotic stress tolerance: A review. Plant Stress 10, 100281. doi: 10.1016/j.stress.2023.100281

[B60] Marques D. N. Carvalho M. E. A. Piotto F. A. Batagin-Piotto K. D. Nogueira M. L. Gaziola S. A.. (2019). “Antioxidant defense response in plants to cadmium stress,” in Cadmium Tolerance in Plants (Oxford OX5 1GB, United Kingdom: Academic Press), 423–461. doi: 10.1016/B978-0-12-815794-7.00016-3

[B61] Maslennikova D. Koryakov I. Yuldashev R. Avtushenko I. Yakupova A. Lastochkina O. (2023). Endophytic plant growth-promoting bacterium *bacillus subtilis* reduces the toxic effect of cadmium on wheat plants. Microorganisms 11, 1653. doi: 10.3390/microorganisms11071653 37512826 PMC10386265

[B62] Masood S. Zhao X. Q. Shen R. F. (2020). *Bacillus pumilus* promotes the growth and nitrogen uptake of tomato plants under nitrogen fertilization. Scientia Hortic. 272, 109581. doi: 10.1016/j.scienta.2020.109581

[B63] Moodie C. Smith H. McCreery R. (1959). Laboratory Manual for Soil Fertility Washington State College (USA: Mimeograph).

[B64] Nafees M. Ali S. Naveed M. Rizwan M. (2018). Efficiency of biogas slurry and Burkholderia phytofirmans PsJN to improve growth, physiology, and antioxidant activity of *Brassica napus* L. @ in chromium-contaminated soil. Environ. Sci. pollut. Res. 25, 6387–6397. doi: 10.1007/s11356-017-0924-z 29249026

[B65] Nafees M. Ali M. Z. Qiu L. L. Yin Y. Xu M. Wang G.. (2024b). Mechanistic approach of tannery wastewater and sulfadiazine mutual toxicity in wheat (*Triticum aestivum* L.) and mitigation through exogenous application of gallic acid. Chemosphere. 358, 142203. doi: 10.1016/j.chemosphere.2024.142203 38697571

[B66] Nafees M. Ali S. Rizwan M. Aziz A. Adrees M. Hussain S. M.. (2020). Effect of nanoparticles on plant growth and physiology and on soil microbes. Nanomater. Environ. Biotechnol. 64 (4), 65–85. doi: 10.1007/978-3-030-34544-0_5

[B67] Nafees M. Sehrish A.k. Alomrani S. A. A. Qiu L. Saeed A. Ahmad S.. (2024a). Mechanism and synergistic effect of sulfadiazine (SDZ) and cadmium toxicity in spinach (*Spinacia oleracea* L.) and its alleviation through zinc fortification. J. Hazard. Mater. 132903, 0304–3894. doi: 10.1016/j.jhazmat.2023.132903 37979422

[B68] Nakano Y. Asada K. (1981). Hydrogen peroxide is scavenged by ascorbate-specific peroxidase in spinach chloroplasts. Plant Cell Physiol. 22, 867–880. doi: 10.1093/oxfordjournals.pcp.a076232

[B69] Nazir M. M. Noman M. Ahmed T. Ali S. Ulhassan Z. Zeng F.. (2022). Exogenous calcium oxide nanoparticles alleviate cadmium toxicity by reducing Cd uptake and enhancing antioxidative capacity in barley seedlings. J. Hazard. Mater. 438, 129498. doi: 10.1016/j.jhazmat.2022.129498 35803196

[B70] Noman M. Ahmed T. Hussain S. Niazi M. B. K. Shahid M. Song F. (2020). Biogenic copper nanoparticles synthesized by using a copper-resistant strain Shigella flexneri SNT22 reduced the translocation of cadmium from soil to wheat plants. J. Hazard. Material. 398, 123175. doi: 10.1016/j.jhazmat.2020.123175 32768848

[B71] Pathania D. Srivastava A. (2020). Advances in nanoparticles tailored lignocellulosic biochars for removal of heavy metals with special reference to cadmium (II) and chromium (VI). Environ. Sustain. 4, 201–214. doi: 10.1007/s42398-020-00142-w

[B72] Rizwan M. Ali S. Ur Rehman M. Z. Rinklebe J. Tsang D. C. Bashir A.. (2018). Cadmium phytoremediation potential of Brassica crop species: a review. Sci. Total Environ. 631, 1175–1191. doi: 10.1016/j.scitotenv.2018.03.104 29727943

[B73] Samborska I. A. Kalaji H. M. Sieczko L. Goltsev V. Borucki W. Jajoo A. (2018). Structural and functional disorder in the photosynthetic apparatus of radish plants under magnesium deficiency. Funct. Plant Biol. 45, 668–679. doi: 10.1071/FP17241 32290968

[B74] Shahzad A. Qin M. Elahie M. Naeem M. Bashir T. Yasmin H.. (2021). Bacillus pumilus induced tolerance of Maize (*Zea mays* L.) against Cadmium (Cd) stress. Sci. Rep. 11, 17196. doi: 10.1038/s41598-021-96786-7 34433897 PMC8387377

[B75] Sharma S. B. Sayyed R. Z. Trivedi M. H. Gobi T. A. (2013). Phosphate solubilizing microbes: sustainable approach for managing phosphorus deficiency in agricultural soils. Springer Plus 2, 1–14. doi: 10.1186/2193-1801-2-587 25674415 PMC4320215

[B76] Shi Z. Carey M. Meharg C. Williams P. N. Signes-Pastor A. J. Triwardhani E. A.. (2020). Rice grain cadmium concentrations in the global supply-chain. Exposure Health 12, 869–876. doi: 10.1007/s12403-020-00349-6

[B77] Song W.-e. Chen S.-B. Liu J.-F. Li C. Song N.-N. Ning L.. (2015). Variation of Cd concentration in various rice cultivars and derivation of cadmium toxicity thresholds for paddy soil by species-sensitivity distribution. J. Integ. Agric. 14, 1845–1854. doi: 10.1016/S2095-3119(14)60926-6

[B78] Srivastava R. K. Pandey P. Rajpoot R. Rani A. Dubey R. (2014). Cadmium and lead interactive effects on oxidative stress and antioxidative responses in rice seedlings. Protoplasma 251, 1047–1065. doi: 10.1007/s00709-014-0614-3 24482190

[B79] Tian S. Xie R. Wang H. Hu Y. Ge J. Liao X.. (2016). Calcium deficiency triggers phloem remobilization of cadmium in a hyperaccumulating species. Plant Physio. 172, 2300–2313. doi: 10.1104/pp.16.01348 PMC5129722 27789737

[B80] Tränkner M. Jákli B. Tavakol E. Geilfus C. M. Cakmak I. Dittert K.. (2016). Magnesium deficiency decreases biomass water-use efficiency and increases leaf water-use efficiency and oxidative stress in barley plants. Plant Soil. 406, 409–423. doi: 10.1007/s11104-016-2886-1

[B81] Ulhassan Z. Bhat J. A. Zhou W. Senan A. M. Alam P. Ahmad P. (2022). Attenuation mechanisms of arsenic induced toxicity and its accumulation in plants by engineered nanoparticles: a review. Environ. pollut. 302, 119038. doi: 10.1016/j.envpol.2022.119038 35196561

[B82] Wang Y. Jiang X. Li K. Wu M. Zhang R. Zhang L.. (2014). Photosynthetic responses of Oryza sativa L. seedlings to cadmium stress: physiological, biochemical and ultrastructural analyses. Biometals 27, 389–401. doi: 10.1007/s10534-014-9720-0 24562500

[B83] Wang Z. Yue L. Dhankher O. P. Xing B. (2020). Nano-enabled improvements of growth and nutritional quality in food plants driven by rhizosphere processes. Environ. Int. 142, 105831. doi: 10.1016/j.envint.2020.105831 32540628

[B84] Wolfe K. Wu X. Liu R. H. (2003). Antioxidant activity of apple peels. J. Agric. Food Chem. 51, 609–614. doi: 10.1021/jf020782a 12537430

[B85] Wu Z. Xu S. Shi H. Zhao P. Liu X. Li F.. (2018). Comparison of foliar silicon and selenium on cadmium absorption, compartmentation, translocation and the antioxidant system in Chinese flowering cabbage. Ecotoxic. Environ. Safety. 166, 157–164. doi: 10.1016/j.ecoenv.2018.09.085 30267988

[B86] Yang S. Gu S. He M. Tang X. Ma L. Q. Xu J.. (2020). Policy adjustment impacts Cd, Cu, Ni, Pb and Zn contamination in soils around e-waste area: Concentrations, sources and health risks. Sci. Total Environ. 741, 140442. doi: 10.1016/j.scitotenv.2020.140442 32615436

[B87] Yang H. Y. Shi G. X. Qiao X. Q. Tian X. L. (2011). Exogenous spermidine and spermine enhance cadmium tolerance of Potamogeton malaianus. Russ. J. Plant Physiol. 58, 622–628. doi: 10.1134/S1021443711040261

[B88] Ye W. Wu F. Zhang G. Fang Q. Lu H. Hu H. (2020). Calcium decreases cadmium concentration in root but facilitates cadmium translocation from root to shoot in rice. J. Plant Growth Regul. 39, 422–429. doi: 10.1007/s00344-019-09992-z

[B89] Yemm E. W. Willis A. (1954). The estimation of carbohydrates in plant extracts by anthrone. Biochem. J. 57, 508. doi: 10.1042/bj0570508 13181867 PMC1269789

[B90] Yu H. Wu Y. Huang H. Zhan J. Wang K. Li T. (2020). The predominant role of pectin in binding Cd in the root cell wall of a high Cd accumulating rice line (*Oryza sativa* L.). Ecotoxicol. Environ. Saf. 206, 111210. doi: 10.1016/j.ecoenv.2020.111210 32890925

[B91] Zeng F. Zahoor M. Waseem M. Anayat A. Rizwan M. Ahmad A.. (2020). Influence of metal-resistant staphylococcus aureus strain K1 on the alleviation of chromium stress in wheat. Agron. 10, 1354. doi: 10.3390/agronomy10091354

[B92] Zhang J. Kirkham M. (1994). Drought-stress-induced changes in activities of superoxide dismutase, catalase, and peroxidase in wheat species. Plant Cell Physiol. 35, 785–791. doi: 10.1093/oxfordjournals.pcp.a078658

[B93] Zhang X. Z. (1992). The measurement and mechanism of lipid peroxidation and SOD, POD and CAT activities in biological system. Res. Method. Crop Physiol., 208–211.

[B94] Zulfiqar U. Ayub A. Hussain S. Waraich E. A. El-Esawi M. A. Ishfaq M.. (2022a). Cadmium toxicity in plants: recent progress on morpho-physiological effects and remediation strategies. J. Soil Sci. Plant Nutr. 22, 212–269. doi: 10.1007/s42729-021-00645-3

[B95] Zulfiqar U. Haider F. U. Ahmad M. Hussain S. Maqsood M.F. Ishfaq M.. (2023a). Chromium toxicity, speciation, and remediation strategies in soil-plant interface: A critical review. Front. Plant Sci. 13. doi: 10.3389/fpls.2022.1081624 PMC9880494 36714741

[B96] Zulfiqar U. Haider F. U. Maqsood M. F. Mohy-Ud-Din W. Shabaan M. Ahmad M.. (2023b). Recent advances in microbial-assisted remediation of cadmium-contaminated soil. Plants 12, 3147. doi: 10.3390/plants12173147 37687393 PMC10490184

[B97] Zulfiqar U. Wenting J. Wang X. Saddam H. Muhammad A. Maqsood M. F.. (2022b). Cadmium phytotoxicity, tolerance, and advanced remediation approaches in agricultural soils; A comprehensive review. Front. Plant Sci. 13, 131664–13462X. doi: 10.3389/fpls.2022.773815 PMC8965506 35371142

